# Neuroprotection and Axonal Regeneration via ECM‐Mimetic Nanofibers Incorporating Metal–Phenolic Network Nanoparticles Toward Spinal Cord Injury Repair

**DOI:** 10.1002/advs.202513825

**Published:** 2025-10-27

**Authors:** Shu Chen, Bixue Wang, Qiya Zhang, Changsheng Liu, Xi Chen

**Affiliations:** ^1^ Key Laboratory for Ultrafine Materials of Ministry of Education Frontiers Science Center for Materiobiology and Dynamic Chemistry Engineering Research Center of Biomedical Materials of Ministry of Education School of Materials Science and Engineering East China University of Science and Technology Shanghai 200237 China

**Keywords:** axonal regeneration, decellularized extracellular matrix, metal–phenolic networks, neuroprotection, spinal cord injury

## Abstract

Spinal cord injury (SCI) triggers secondary pathological cascades, such as excitotoxicity, neuroinflammation, and glial scarring, creating a hostile injury microenvironment that exacerbates the death of spared neurons and inhibits axonal regeneration, thus impeding functional recovery. While aligned electrospun nanofibers (NFs) fabricated from decellularized extracellular matrix (dECM) can mimic topological and bioactive cues of native ECM to guide axonal regrowth, they fail to intervene in these secondary pathological cascades. To address this limitation, Mg^2+^ and epigallocatechin gallate (EGCG) molecules are initially self‐assembled into nanoparticles (MPN NPs) via metal–phenolic coordination, and then integrated with dECM by electrospinning to form an aligned fibrous scaffold, MPN@dECM NFs. These MPN NPs undergo pH‐responsive degradation in the acidic SCI microenvironment, releasing Mg^2+^ to alleviate excitotoxicity by blocking Ca^2+^ influx and EGCG to suppress neuroinflammation by reducing pro‐inflammatory mediators. In the mouse SCI model, MPN@dECM NFs consistently attenuate neuroinflammation and glial scarring, and ameliorate the regeneration‐inhibitory microenvironment, thereby not only protecting spared neurons from secondary degeneration but also enhancing dECM‐mediated axonal regeneration. Ultimately, surviving neurons and regenerating axons synergize to facilitate motor function restoration. Collectively, this scaffold design synergistically promotes neuroprotection and axonal regeneration, demonstrating enhanced repair efficacy for SCI as a promising therapeutic strategy.

## Graphical abstract

Metal–phenolic network (MPN) nanoparticles with neuroprotective functions are integrated into decellularized extracellular matrix (dECM) to obtain biomimetic composite fibrous scaffold MPN@dECM NFs by electrospinning, which protects spared neurons from secondary degeneration by suppressing excitotoxicity and inflammation, and promotes directional regeneration of axons, thus synergistically restoring motor functions after spinal cord injury.

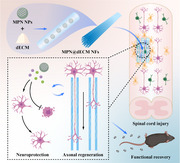

## 1 Introduction

Spinal cord injury (SCI) poses a critical global health burden, with epidemiologic studies reporting alarming annual increases in incidence, prevalence, and years lived with disability (YLDs), currently affecting ≈20 million patients worldwide.^[^ ^1^, ^2^ ^]^ However, effective clinical interventions remain elusive due to the complex pathophysiology of SCI, where primary mechanical trauma initiates progressive secondary neurodegeneration. This degenerative cascade, driven by excitotoxicity, neuroinflammation, and reactive astrocyte activation, exacerbates spared neuronal death, axonal degeneration, and demyelination. Critically, these spatially and temporally overlapping pathologies consolidate into a self‐sustaining inhibitory microenvironment that actively blocks regenerative processes.^[^ ^3^, ^4^ ^]^ Consequently, therapeutic strategies that counteract this hostile microenvironment while enabling concurrent neuroprotection and axonal regeneration represent an urgent unmet need.^[^ ^5^ ^]^

Decellularized extracellular matrix (dECM) scaffolds retain tissue‐specific topology, biomechanical properties, and bioactive signals, establishing them as premier biomimetic platforms for neural repair.^[^ ^6^, ^7^, ^8^ ^]^ For instance, the retained ECM glycoprotein laminin in dECM plays a key role in cell adhesion, migration, and differentiation, and it has been demonstrated to promote the development of the oligodendroglial lineage.^[^ ^9^, ^10^, ^11^ ^]^ Additionally, the retained structural ECM component collagen can be fabricated into a scaffold to limit glial scar deposition, guide axon elongation, and maintain neuronal survival in SCI.^[^ ^12^, ^13^ ^]^ Significantly, spinal cord‐derived dECM specifically preserves neuroregenerative cues that promote neural stem cell migration, axonal regeneration, and functional recovery in SCI rodent models.^[^ ^10^, ^14^ ^]^ Electrospinning further engineers these scaffolds into aligned nanofibers (NFs) that recapitulate spinal cord ECM anisotropy, thereby enhancing directional axonal guidance.^[^ ^11^ ^]^ However, current studies predominantly emphasize the provision of biochemical signals and structural support for regeneration, while paying insufficient attention to ongoing neurodegeneration and microenvironmental inhibition. This limitation potentially results in suboptimal structural regeneration and functional recovery of the spinal cord. Therefore, to achieve comprehensive functional recovery after SCI, next‐generation dECM scaffolds need to integrate neuroprotective functionalities to address the complex pathological processes of SCI.

Following acute SCI, vascular rupture or compression at the lesion site induces a marked reduction in local blood flow. Subsequent ischemia–hypoxia‐driven acidosis reduces extracellular pH to 6.4–6.7, triggering a cascade of deleterious events.^[^ ^15^, ^16^ ^]^ On one hand, the acidic conditions recruit and activate immune cells (e.g., microglia, macrophages), and stimulate these inflammatory cells to secrete abundant pro‐inflammatory cytokines and chemokines, amplifying local inflammation.^[^ ^15^, ^17^ ^]^ This inflammation, in turn, further exacerbates lesion‐site acidosis,^[^ ^18^, ^19^ ^]^ establishing an “acidosis–inflammation” vicious cycle. On the other hand, the acidic conditions enhance the activity of N‐methyl‐D‐aspartate (NMDA) ionotropic glutamate receptors during the subacute phase of SCI,^[^ ^20^, ^21^ ^]^ inducing Ca^2+^ influx and subsequent intracellular Ca^2+^ overload, ultimately increasing intracellular reactive oxygen species (ROS) levels and exacerbating neuronal excitotoxicity.^[^ ^22^, ^23^ ^]^ Metal–phenolic networks (MPNs) are supramolecular networks formed by the coordination of metal ions and ligands containing catechol moieties.^[^ ^24^, ^25^, ^26^ ^]^ Due to their pH‐dependent reversible self‐assembly process,^[^ ^27^, ^28^ ^]^ they are predictably positioned for pH‐responsive neuroprotective cargo delivery in the acidic microenvironment of SCI. The physiological functions of MPNs are intrinsically governed by the constituent metal ions and phenol ligands, enabling programmable functionality design. Particularly, functioning as a physiological NMDA receptor antagonist, Mg^2+^ selectively blocks glutamate‐gated Ca^2+^ channels to mitigate neuronal excitotoxicity under pathological conditions.^[^ ^29^, ^30^ ^]^ Epigallocatechin gallate (EGCG) exerts potent neuroprotective effects in central nervous system (CNS) disorders via antioxidant, anti‐inflammatory actions and suppression of reactive astrogliosis.^[^ ^31^, ^32^, ^33^, ^34^ ^]^ Therefore, MPNs composed of Mg^2+^ and EGCG are expected to possess dual protective modalities in inhibiting excitotoxicity and neuroinflammation, thereby mitigating secondary neurodegeneration and remodeling the microenvironment to facilitate axonal regeneration after SCI.

**Scheme 1:**
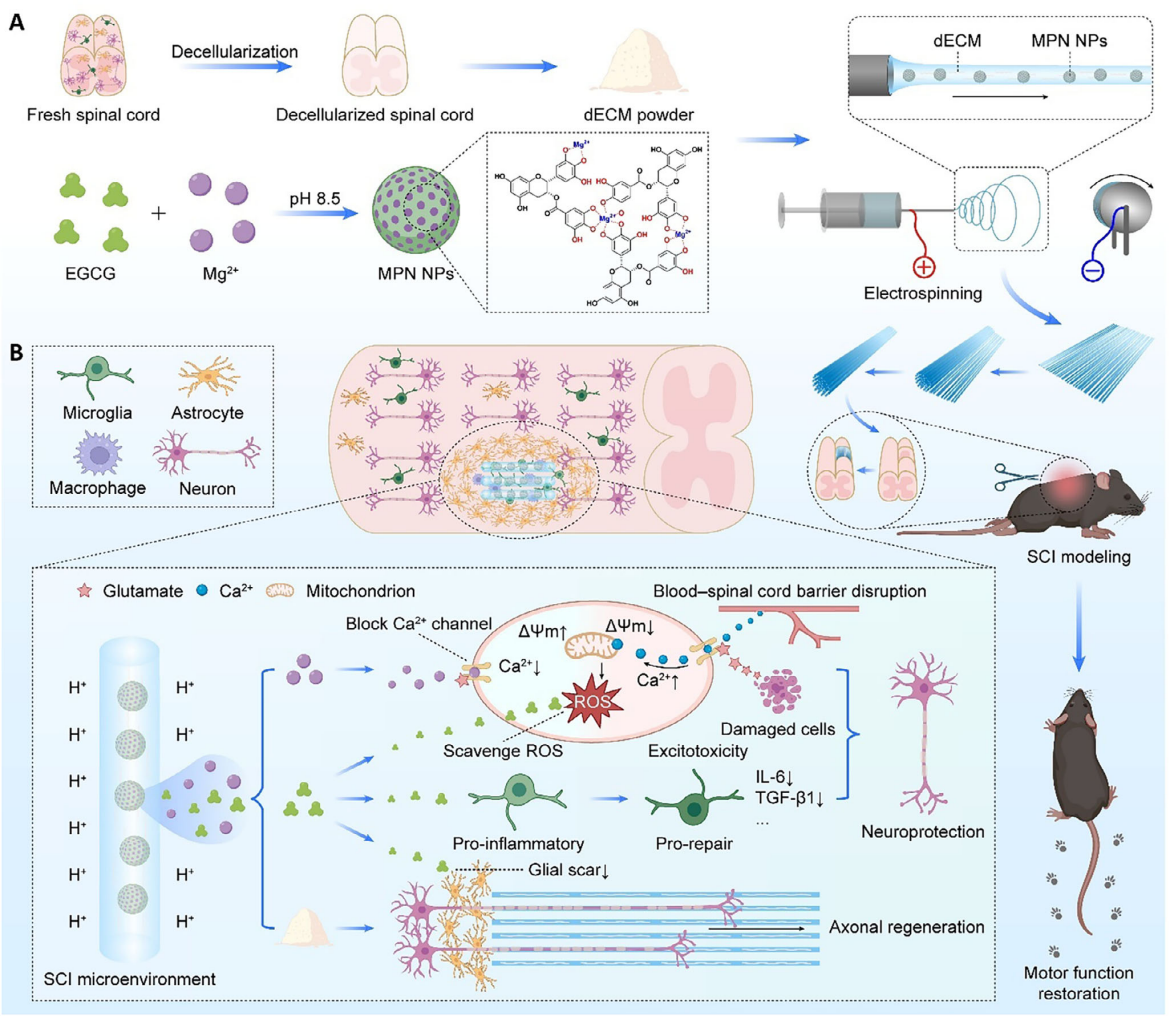
Schematic illustration of the fabrication and therapeutic mechanisms of MPN@dECM NFs for the treatment of SCI. A) dECM and Mg–EGCG MPN nanoparticles (NPs) were prepared and synthesized, respectively, and then integrated via co‐electrospinning into an aligned fibrous scaffold, MPN@dECM NFs. B) After implantation of MPN@dECM NFs, acidic SCI microenvironment‐triggered release of Mg^2+^ and EGCG protected spared neurons via attenuating excitotoxicity and suppressing neuroinflammation, while dECM fibers with aligned topographic structure directed axonal regeneration and remyelination, ultimately restoring motor function after SCI.

Herein, to construct a multifunctional scaffold integrating neuroprotection and axonal regeneration functions for SCI repair, spinal cord‐derived dECM and Mg–EGCG MPN nanoparticles (NPs) were pre‐prepared, and then composited by co‐electrospinning to form a directionally aligned composite fibrous scaffold, MPN@dECM NFs (**Scheme** **1**A). As the nanofibers undergo degradation at the implantation site, MPN NPs are progressively exposed. These MPN NPs respond to the acidic SCI microenvironment, where the protonation of the catechol groups in EGCG triggers their disassembly into Mg^2+^ and EGCG,^[^ ^35^ ^]^ which subsequently act to inhibit excitotoxicity and neuroinflammation. Simultaneously, the scaffold sustains the neurotrophic support of dECM while providing topographic guidance through its ECM‐mimetic architecture, thereby promoting axonal regeneration, neuronal orientation, and ultimate functional recovery post‐SCI (Scheme 1B). Cellular experiments demonstrated that MPN@dECM NFs robustly inhibited excitotoxic neuronal death by suppressing Ca^2+^ overload, mitochondrial depolarization, and oxidative damage. Concurrently, the scaffold potently attenuated neuroinflammation by suppressing pro‐inflammatory gene expression in microglia, reducing pro‐inflammatory cytokine secretion, and promoting their polarization toward a pro‐repair phenotype. Then, its therapeutic effect on SCI was comprehensively evaluated using a mouse spinal cord hemisection model. MPN@dECM NFs significantly suppressed glial scarring, attenuated degeneration of spared neurons, promoted axonal regeneration and remyelination at the lesion site, and ultimately restored hindlimb locomotor function and weight‐bearing capacity in SCI mice. The neuroprotective efficacy of MPN@dECM NFs substantially potentiated axonal regrowth and functional recovery, underscoring the therapeutic promise of this dual‐pathway strategy that integrates neuroprotection with axonal regeneration.

## 2 Results and Discussion

### 2.1 Preparation and Characterization of dECM

**Figure 1:**
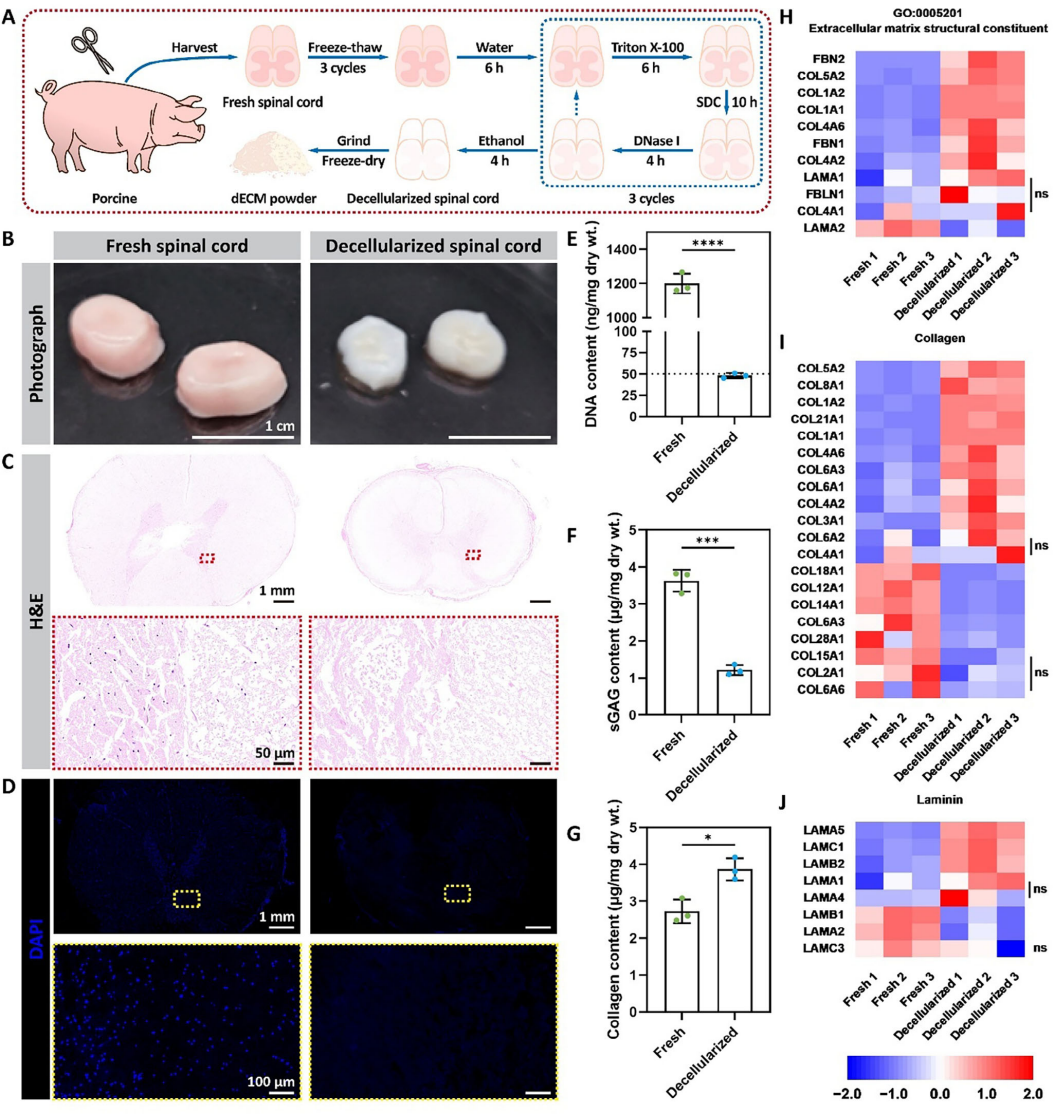
Preparation and characterization of dECM. A) Scheme of the decellularization process of the porcine spinal cord. B) Photographs of fresh and decellularized spinal cord segments. Scale bar, 1 cm. C,D) Images of H&E (C) or DAPI (D) staining of fresh and decellularized spinal cord cross‐sections. Scale bar, 1 mm in low‐magnification images, 50 µm (C) or 100 µm (D) in high‐magnification images. E–G) Quantification of DNA (E), sGAG (F), or Collagen (G) contents of fresh and decellularized spinal cord. Data were presented as mean ± SD (*n* = 3). Statistical differences were determined using a two‐tailed unpaired *t*‐test (* *p* < 0.05, *** *p* < 0.001, **** *p* < 0.0001). H–J) Heatmaps of contents of proteins annotated with GO:0005201 (extracellular matrix structural constituent) (H), collagen (I), or laminin (J) in fresh and decellularized spinal cord by proteomic analysis. Protein contents were presented using Z‐score. Statistical differences were determined using *t*‐test, and proteins with |log2FC| > 0.263 and *p* < 0.05 were considered significantly different (ns indicates no significance).

Due to the wide availability of organs and tissues, porcine is currently one of the main source species for dECM.^[^ ^7^ ^]^ To prepare porcine spinal cord‐derived dECM, we applied a decellularization protocol,^[^ ^36^ ^]^ which combines freeze–thaw cycles, detergent treatments, and deoxyribonuclease (DNase) treatments (**Figure** **1**A). After decellularization, the spinal cord segments faded from pink to white color and decreased in size while retaining morphology (Figure 1B). To test the effect of decellularization, decellularized spinal cord segments were stained with hematoxylin and eosin (H&E). The images showed that hematoxylin‐positive nuclei were barely observed after decellularization, while eosin‐positive proteins were largely retained (Figure 1C). DAPI staining further demonstrated that the nuclei were largely removed after decellularization (Figure 1D). Next, the content of DNA, sulfated glycosaminoglycans (sGAG), and collagen in the decellularized spinal cord was examined. The results showed that the DNA content decreased from 1200.2 ± 58.0 to 48.0 ± 2.8 ng mg^−1^ with a DNA removal rate of 96.0% (Figure 1E), which meets the standard criteria for decellularization.^[^ ^37^ ^]^ The sGAG content decreased from 3.63 ± 0.30 to 1.21 ± 0.13 µg mg^−1^ (Figure 1F), while the collagen content increased from 2.73 ± 0.32 to 3.87 ± 0.30 µg mg^−1^ (Figure 1G).

To understand the protein composition after decellularization, proteomic analysis was performed. A Pearson correlation coefficient (PCC) heatmap revealed marked compositional alterations post‐decellularization (PCC 0.61–0.67 vs. fresh samples) but preserved intra‐group homogeneity (intra‐group PCC ≥ 0.96) (Figure S1A, Supporting Information). The volcano plot identified 1565 upregulated and 2135 downregulated proteins (|log2FC| > 0.263, *p* < 0.05) post‐decellularization, with 3302 proteins showing no significant alteration (Figure S1B, Supporting Information). A principal component analysis (PCA) showed clear separation between decellularized and fresh samples along PCA1 (68.34% variance explained; reflecting decellularization‐induced compositional changes) and within the decellularized group along PCA2 (10.39% variance; indicating minor batch effects) (Figure S1C, Supporting Information). Differentially expressed proteins (DEPs, ranked by *p*‐values) heatmap revealed decellularization‐induced compositional changes (Figure S1D, Supporting Information). Focusing on DEPs annotated with the Gene Ontology (GO) term GO:0005576 (extracellular region), key structural components—including collagen (COL), laminin (LAM), elastin (ELN), fibrillin (FBN), and fibulin (FBLN)—showed predominant upregulation (e.g., COL6A1/6A3, LAMC1, FBN1/2) with limited downregulation (e.g., COL12A1, LAMA2) (Figure S1E, Supporting Information). Further analysis of ECM structural constituents (GO:0005201), collagens, and laminins confirmed this upregulation trend (Figure 1H–J). These spinal cord‐specific ECM structural proteins play a non‐negligible role in enabling the dECM to exert a positive effect on promoting axonal regrowth and inducing differentiation of neural stem/progenitor cells in SCI.^[^ ^14^, ^38^, ^39^, ^40^, ^41^, ^42^, ^43^, ^44^, ^45^, ^46^, ^47^ ^]^ Collectively, these results indicate the successful preparation of dECM, characterized by effective removal of immunogenic DNA alongside substantial retention of ECM structural components, which are critical for orchestrating endogenous regenerative processes.^[^ ^48^ ^]^

### 2.2 Synthesis and Characterization of MPN NPs

**Figure 2:**
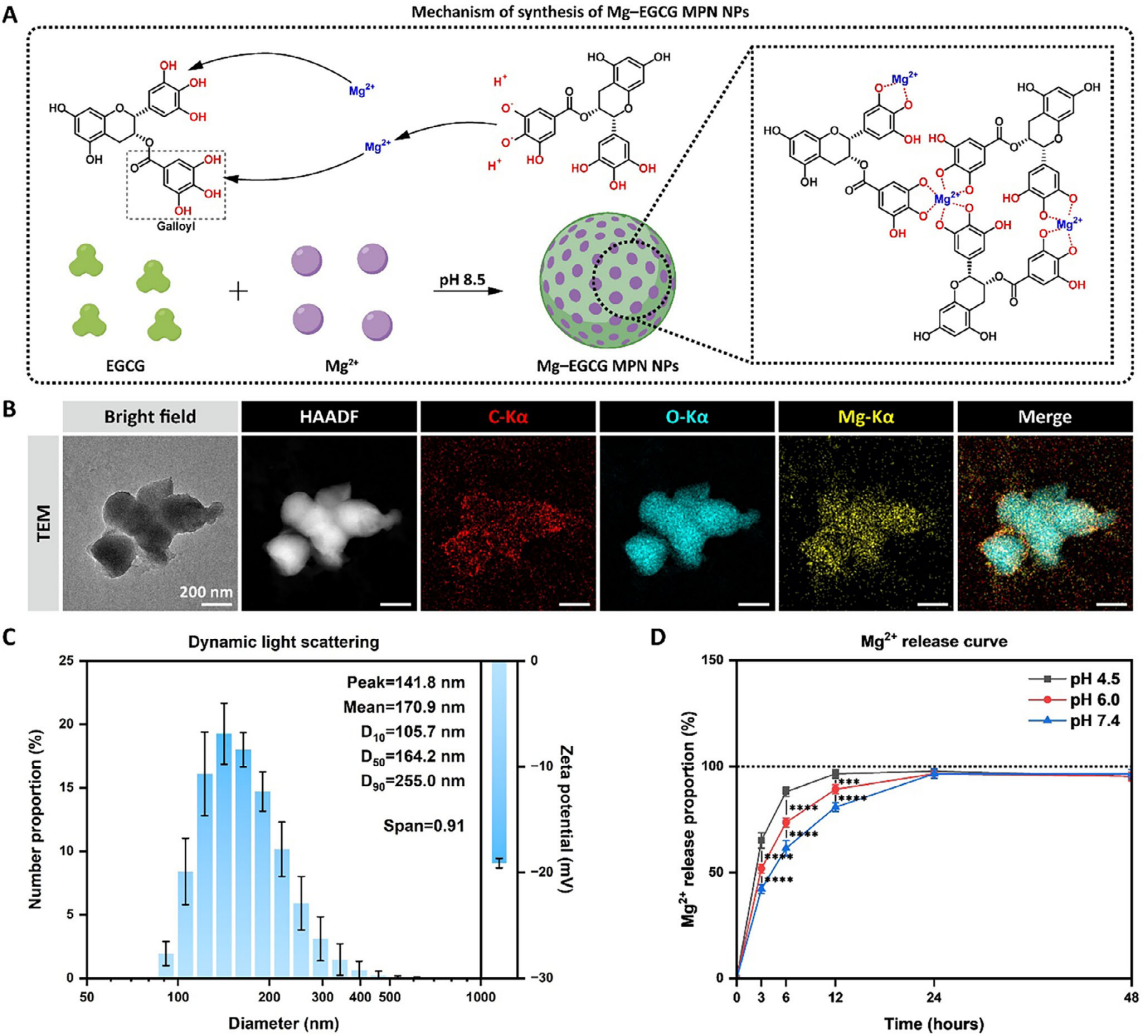
Synthesis and characterization of MPN NPs. A) Scheme of the synthesis mechanism of Mg–EGCG MPN NPs. B) TEM images under bright‐field and HAADF‐STEM modes with corresponding EDS elemental mapping (C‐Kα, O‐Kα, Mg‐Kα) of Mg–EGCG MPN NPs. Scale bar, 200 nm. C) Hydrodynamic diameter distribution by DLS and zeta potential of Mg–EGCG MPN NPs. D) pH‐dependent Mg^2+^ release curves of Mg–EGCG MPN NPs. Data were presented as mean ± SD (*n* = 3 (C,D)). Statistical differences were determined using two‐way ANOVA (D) with Tukey's post hoc test (*** *p* < 0.001, **** *p* < 0.0001).

Meanwhile, Mg–EGCG MPN NPs were synthesized in ethanol through alkaline‐driven coordination (pH 8.5), where the deprotonated catechol groups of EGCG self‐assembled with Mg^2+^ into MPN NPs (**Figure** **2**A), as indicated by the distinct color change to purple‐brown (Figure S2, Supporting Information). To validate the synthesis and evaluate the properties, the morphology, size distribution, surface charge, chemical composition, and pH‐responsive release behavior of the NPs were systematically characterized.

Transmission electron microscopy (TEM) imaging revealed quasi‐spherical MPN NPs with irregular surfaces, while energy dispersive X‐ray spectroscopy (EDS) elemental mapping confirmed homogeneous distribution of C, O, and Mg throughout the NPs (Figure 2B). Dynamic light scattering (DLS) analysis revealed a narrow size distribution of the NPs, characterized by a peak hydrodynamic diameter of 141.8 nm, a mean diameter of 170.9 nm, and a median diameter of 164.2 nm, with a *Span* value (defined as (*D*_90_−*D*_10_)/*D*_50_) of 0.91 (Figure 2C). This high monodispersity is critical for maintaining stability during the electrospinning process, thereby facilitating uniform NP dispersion within fibers.^[^ ^49^ ^]^ In addition, the surface zeta potential of NPs in water was −19.1 ± 0.5 mV, suggesting favorable dispersion stability and compatibility with polar solvents. Fourier transform infrared spectroscopy (FTIR) confirmed the formation of the Mg–EGCG coordination network, showing characteristic shifts in phenolic hydroxyl and carbonyl stretching vibrations consistent with metal ion coordination and structural reorganization.^[^ ^50^ ^]^ A distinct Mg─O peak at 478 cm^−1^ further supported coordination bond formation (Figure S3A, Supporting Information).^[^ ^51^ ^]^ X‐ray photoelectron spectroscopy (XPS) analysis verified the elemental composition (C: 63.46%, O: 32.62%, Mg: 3.92%; C:O ≈ 2:1), matching EGCG stoichiometry and indicating minimal inorganic Mg contaminants. The Mg‐KLL Auger peak position (306 eV) was characteristic of Mg─O coordination bonds within the MPNs (Figure S3B, Supporting Information). These data collectively confirm the successful synthesis of Mg–EGCG MPN NPs.

Inductively coupled plasma optical emission spectroscopy (ICP‐OES) confirmed the pH‐responsive Mg^2+^ release from Mg–EGCG MPN NPs (Figure 2D). An initial rapid release occurred within 3 h (pH 4.5: 65.1%; pH 6.0: 51.9%; pH 7.4: 42.2%), followed by sustained release. The most pronounced between‐group difference was observed within 6 h (pH 4.5: 88.1%; pH 6.0: 73.6%; pH 7.4: 61.5%), with lower pH conditions showing significantly higher release rates. The enhanced release profiles at pH 6.0 (mimicking the acidic SCI microenvironment) and 4.5 (mimicking the lysosomal microenvironment) indicate that the NPs undergo accelerated release in the SCI microenvironment while being rapidly metabolized following cellular internalization, which aligns with our design objectives.

### 2.3 Fabrication and Characterization of MPN@dECM NFs

**Figure 3:**
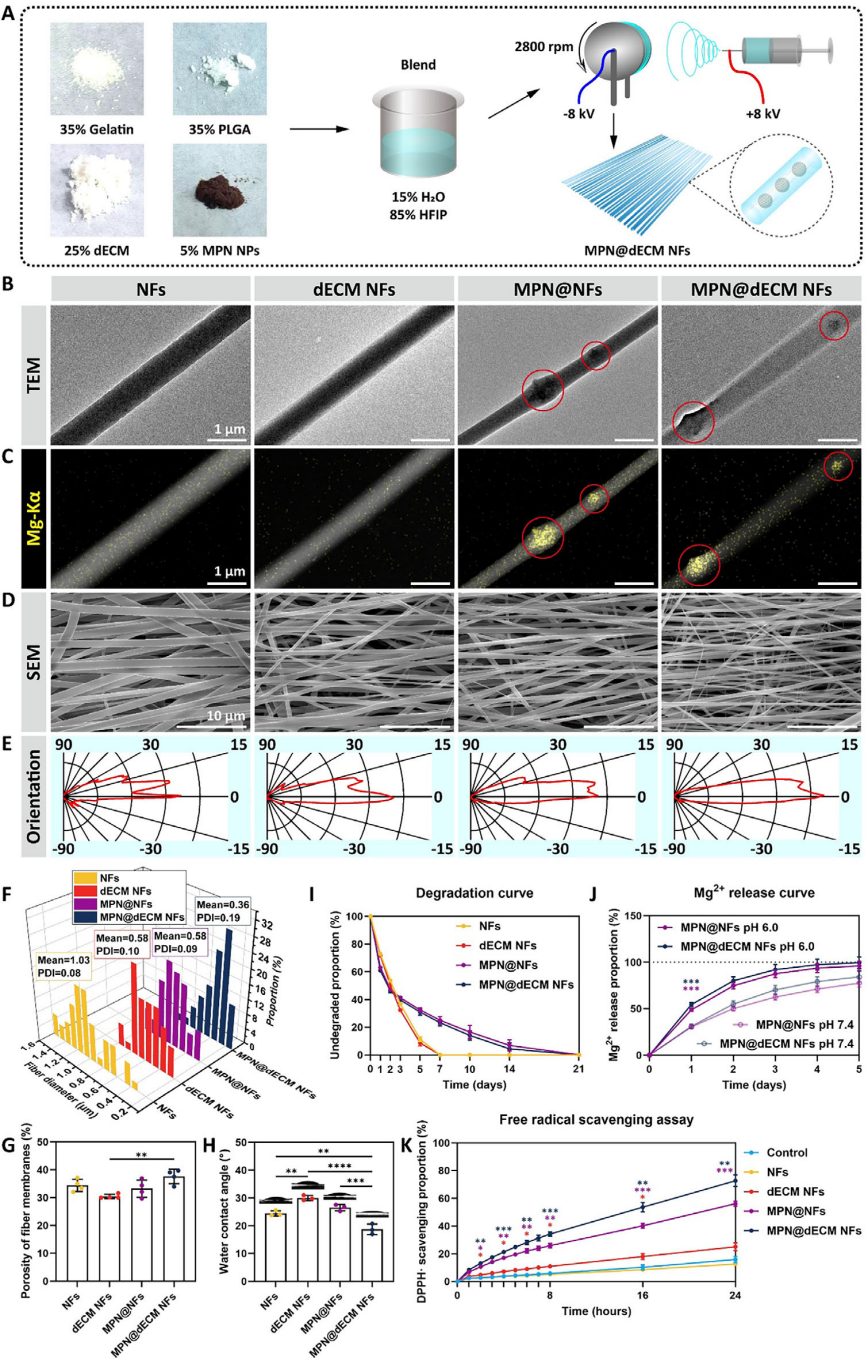
Fabrication and characterization of MPN@dECM NFs. A) Scheme of fabrication of MPN@dECM NFs. B,C) TEM images under bright‐field (B) and HAADF‐STEM modes with elemental mapping (Mg‐Kα) (C) of fibers, with MPN NPs highlighted by red circles. Scale bar, 1 µm. D) SEM micrographs of fiber membranes. Scale bar, 10 µm. E) Fiber orientation distribution analyzed by ImageJ. F) Fiber diameter distribution quantified from SEM images (50 random fibers were analyzed). G) Porosity of fiber membranes analyzed by ImageJ. H) Water contact angles of fiber membranes. I) Degradation curves of fiber membranes in saline (pH 6.0, 37 °C) based on area reduction. J) pH‐dependent Mg^2+^ release curves of MPN‐containing fiber membranes, with significant differences between different pH. K) DPPH free radical scavenging by fiber membranes over time. Data were presented as mean ± SD (*n* = 4 (G), *n* = 3 (H–K)). Statistical differences were determined using one‐way ANOVA (G,H) or two‐way ANOVA (J,K) with Tukey's post hoc test (* *p* < 0.05, ** *p* < 0.01, *** *p* < 0.001, **** *p* < 0.0001).

To replicate the aligned architecture of the native spinal cord, we fabricated directionally aligned nanofibers via electrospinning, combining 25 wt.% spinal cord‐derived dECM (to endow bioactive signals), 35 wt.% PLGA (for mechanical stability), 35 wt.% gelatin (to enhance hydrophilicity and cell interaction), and 5 wt.% MPN NPs (for pH‐responsive therapeutic release) (**Figure** **3**A). In order to systematically investigate the influence of individual components on fiber structure, properties, and biological effects, while evaluating their synergistic effects, four scaffolds were fabricated for comparative analysis: NFs (PLGA/gelatin only), dECM NFs (incorporating 25 wt.% dECM with PLGA/gelatin), MPN@NFs (integrating 5 wt.% MPN NPs into PLGA/gelatin), and MPN@dECM NFs (combining 25 wt.% dECM and 5 wt.% MPN NPs with PLGA/gelatin) (Figure S4, Supporting Information).

To characterize the microscopic morphology and structure of MPN@dECM NFs, TEM and scanning electron microscopy (SEM) were employed. TEM imaging revealed the presence of well‐dispersed, high‐contrast NPs within the nanofibers (Figure 3B), which was further confirmed by EDS elemental mapping (Figure 3C; Figure S5, Supporting Information). SEM confirmed that all scaffolds formed oriented, aligned fiber structures with uniform diameters (Figure 3D). The introduction of dECM and MPN NPs improved fiber orientation (Figure 3E) and reduced fiber diameter (Figure 3F). Among the four groups, MPN@dECM NFs exhibited the optimal orientation and the minimal fiber diameter (0.36 µm). Porosity measurements revealed a significant increase in the porosity of MPN@dECM NFs compared to dECM NFs (Figure 3G). These morphological and structural features, including excellent orientation, micro‐ and nanoscale diameters, and favorable porosity, mimic the natural spinal cord ECM topographic structure, which is expected to guide axonal orientation and facilitate nutrient exchange during regeneration.

To evaluate hydrophilicity, water contact angles (CA) were measured, and the results revealed superior wettability across all fiber membrane groups (Figure 3H). The CA trend strongly correlated with porosity, with MPN@dECM NFs achieving optimal hydrophilicity (CA = 18.8 ± 1.9°), primarily driven by capillary effects from micro/nanofiber gaps. Degradation kinetics in the simulated SCI microenvironment (pH 6.0) revealed accelerated initial degradation for MPN@NFs (63.0% residual area) and MPN@dECM NFs (61.0%) versus NFs (72.9%) and dECM NFs (71.9%) at Day 1 (Figure 3I). However, subsequent degradation rates of MPN‐modified fibers decreased substantially, which may be attributed to hydrogen bond‐mediated physical crosslinking formed between residual MPNs—particularly their EGCG components—and both gelatin and PLGA within the fiber matrix. While NFs and dECM NFs fully degraded by Day 7, MPN@NFs and MPN@dECM NFs required more than 14 days for complete degradation, thereby providing extended structural support for chronic‐phase tissue repair. Furthermore, Mg^2+^ release profiles of the fiber membranes indicated that, compared to bare MPN NPs (Figure 2D), the degradation and release of MPN NPs incorporated within the nanofibers were significantly prolonged, while still retaining pH‐responsive release behavior (Figure 3J). Specifically, sustained Mg^2+^ release was achieved over 3–5 days under the mildly acidic condition (pH 6.0). To evaluate the antioxidant capacity of MPN@dECM NFs, a DPPH radical scavenging assay was performed. At 24 h, MPN@dECM NFs demonstrated the highest scavenging efficiency (72.7%), followed by MPN@NFs (56.1%)—both substantially exceeding the Control group (non‐scaffold, 15.9%) (Figure 3K). While dECM NFs showed transient antioxidant activity (e.g., at 16 h), attributable to residual ECM components like collagen,^[^ ^52^ ^]^ the sustained radical scavenging of MPN‐scaffolds primarily originated from EGCG. This potent antioxidant capacity of MPN@dECM NFs is anticipated to confer critical neuroprotection against oxidative damage and attenuate neuroinflammation during SCI repair.

To validate the biosafety of MPN@dECM NFs for therapeutic application, the cytocompatibility was confirmed using HT‐22 neurons, a mouse hippocampal neuronal cell line (Figure S6A, Supporting Information). Cell Counting Kit (CCK)‐8 assessment revealed that while gelatin‐based NFs moderately enhanced viability on days 3–5 (likely attributable to gelatin's pro‐proliferative effect), all scaffold groups (dECM NFs, MPN@NFs, MPN@dECM NFs) maintained viability comparable to the Control group (non‐scaffold) throughout cultivation (Figure S6B, Supporting Information), demonstrating the overall biosafety. Moreover, cells seeded on all fiber membranes exhibited cytoskeletal alignment and elongation along the fiber direction, indicating the excellent capability of the scaffolds to promote cell adhesion and guided orientation (Figure S7, Supporting Information), which is particularly beneficial for guiding axonal outgrowth during spinal cord repair.

### 2.4 MPN@dECM NFs Alleviated Excitotoxicity In Vitro

**Figure 4:**
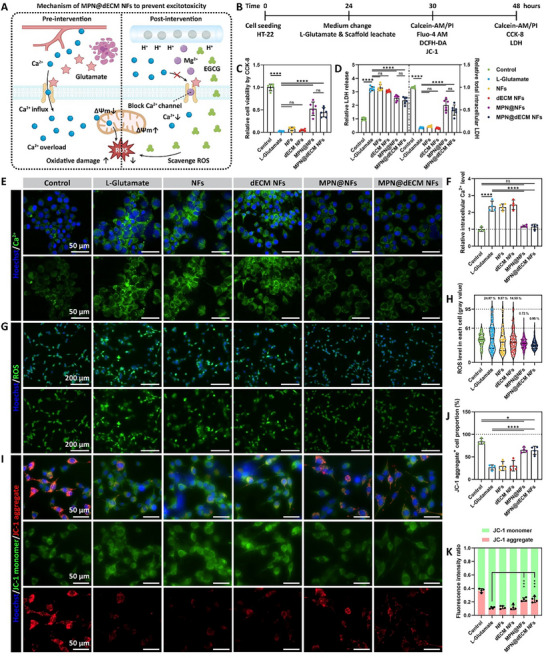
MPN@dECM NFs alleviated excitotoxicity in vitro. A) Schematic mechanism of MPN@dECM NFs to prevent glutamate‐induced excitotoxicity. B) Experimental workflow for in vitro excitotoxicity inhibition assay. C) Relative cell viability assessed by CCK‐8 assay. D) Relative extracellular LDH release levels and total intracellular LDH levels. E,F) Images (E) and quantification (F) of fluorescence staining of intracellular Ca^2+^ levels (Fluo‐4 AM). Scale bar, 50 µm. G,H) Images (G) and quantification (H) of fluorescence staining of intracellular ROS levels (DCFH‐DA). Scale bar, 200 µm. I–K) JC‐1 mitochondrial membrane potential fluorescence staining (I) and quantification of the proportion of JC‐1 aggregate‐positive cells (J) and JC‐1 aggregate‐to‐monomer ratio (K). Scale bar, 50 µm. Data were presented as mean ± SD (*n* = 6 (C,D), *n* = 4 (F,J,K)) or as violin plot (H). Statistical differences were determined using one‐way ANOVA with Tukey's post hoc test (ns indicates no significance, * *p* < 0.05, *** *p* < 0.001, **** *p* < 0.0001).

The incorporation of MPN NPs into the composite scaffold (MPN@dECM NFs) was designed to combat excitotoxicity via pH‐responsive co‐release of Mg^2+^ and EGCG in acidic SCI microenvironments. Mg^2+^ blocks Ca^2+^ influx through NMDA receptors to avoid Ca^2+^ overload, thereby alleviating oxidative damage and maintaining mitochondrial homeostasis, whereas EGCG provides complementary antioxidant protection against ROS (**Figure** **4**A). To assess this neuroprotective function, HT‐22 neurons were exposed to the leachate of MPN@dECM NFs under the condition of L‐Glutamate‐induced excitotoxicity (Figure 4B). First, cell viability was measured to evaluate the neuroprotective effect of MPN@dECM NFs against excitotoxicity. CCK‐8 assay showed that neuronal viability plummeted to 0.02‐fold of Control (untreated medium) after L‐Glutamate treatment for 24 h. There was no significant difference between the NFs and dECM NFs groups and the L‐Glutamate group, whereas MPN@NFs (0.52‐fold) and MPN@dECM NFs (0.45‐fold) significantly restored viability (Figure 4C). Lactate dehydrogenase (LDH) assay showed that extracellular LDH in the L‐Glutamate group elevated to 3.24‐fold of Control, indicating cell membrane rupture, while intracellular LDH decreased to a minimal level (Figure 4D). MPN@NFs (2.55‐fold) and MPN@dECM NFs (2.37‐fold) significantly reduced LDH release while partially restoring intracellular LDH levels, which was consistent with the trend of CCK‐8 results. Live/dead cell staining further demonstrated the neuroprotective effect of MPN@NFs and MPN@dECM NFs against excitotoxicity in a high‐concentration glutamate environment, achieving almost total neuronal survival at 6 h (Figure S8, Supporting Information).

To further investigate the effect of MPN@dECM NFs on excitotoxicity, we performed fluorescent staining for Ca^2+^, ROS, and JC‐1 mitochondrial membrane potential in HT‐22 neurons after 6 h glutamate treatment. Ca^2+^ staining showed that glutamate treatment increased the intracellular Ca^2+^ level to 2.37‐fold of Control, whereas MPN@NFs (1.16‐fold) and MPN@dECM NFs (1.15‐fold) effectively suppressed Ca^2+^ overload, returning levels nearly to baseline (Figure 4E,F). ROS staining showed that glutamate induction resulted in 24.87% of ROS‐positive cells, and MPN@NFs (0.72%) and MPN@dECM NFs (0.95%) reduced the ROS nearly to the Control level (Figure 4G,H), suggesting alleviated oxidative damage. JC‐1 mitochondrial membrane potential staining showed that glutamate treatment reduced the proportion of JC‐1 aggregate‐positive cells (with normal membrane potential) from 84.5% (Control) to 26.6%, whereas MPN@NFs (65.4%) and MPN@dECM NFs (64.5%) partially restored the mitochondrial membrane potential, suggesting the mitigation of the mitochondrial damage and the maintenance of homeostasis (Figure 4I–K). Collectively, these results indicate that MPN@dECM NFs and MPN@NFs multidimensionally inhibit the excitotoxic cascade responses, including Ca^2+^ overload, oxidative damage, and mitochondrial depolarization, thereby protecting neurons exposed to high glutamate concentrations, which is essential for preventing neurodegeneration after SCI.^[^ ^53^, ^54^, ^55^ ^]^

### 2.5 MPN@dECM NFs Suppressed Inflammation In Vitro

**Figure 5:**
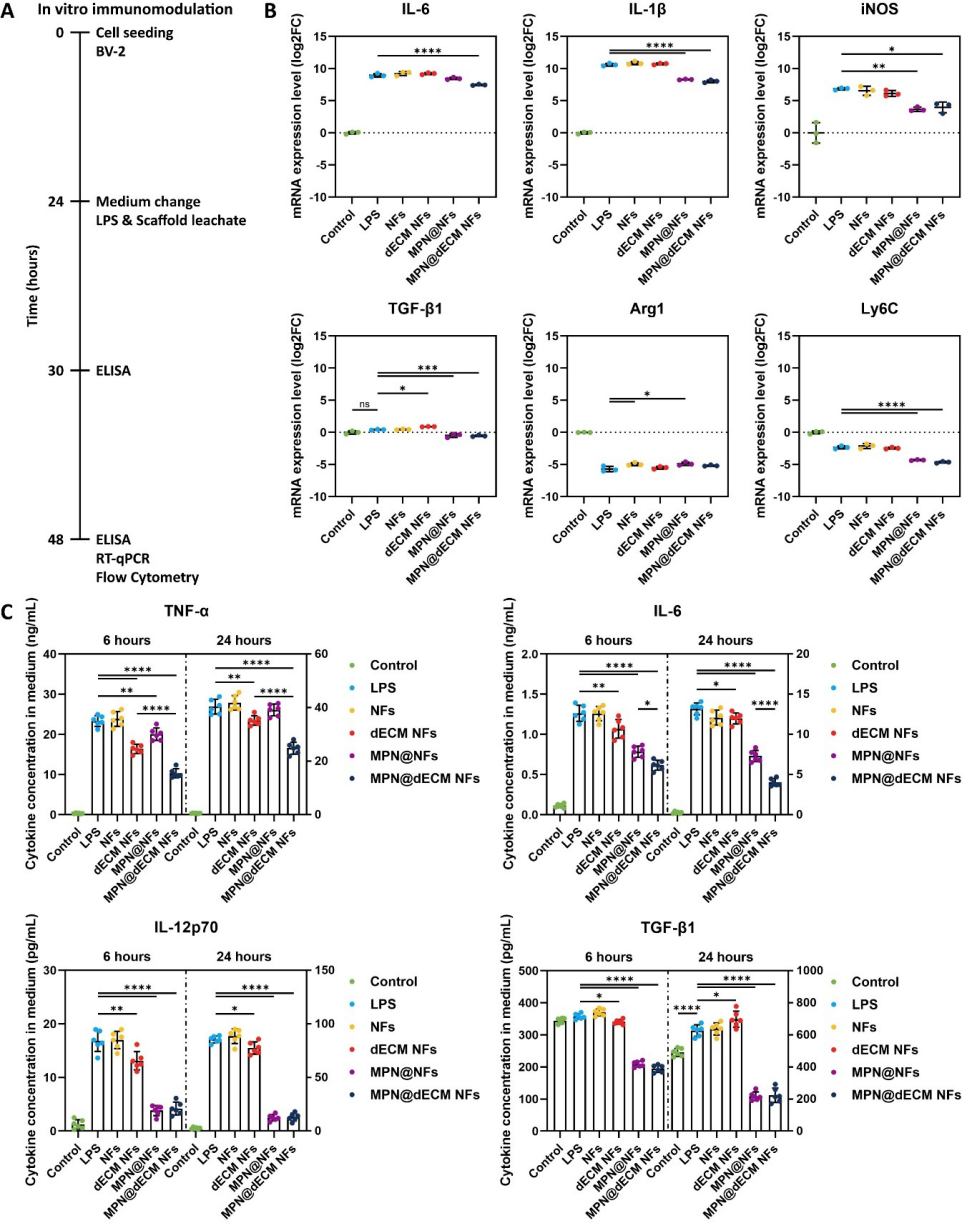
MPN@dECM NFs suppressed inflammation in vitro. A) Experimental workflow for in vitro immunomodulation assay. B) Quantification of mRNA expression of IL‐6, IL‐1β, iNOS, TGF‐β1, Arg1 and Ly6C by RT–qPCR (log2FC). C) Quantification of cytokine levels of TNF‐α, IL‐6, IL‐12p70, and TGF‐β1 by ELISA. Data were presented as mean ± SD (*n* = 3 (B), *n* = 6 (C)). Statistical differences were determined using one‐way ANOVA with Tukey's post hoc test (ns indicates no significance, * *p* < 0.05, ** *p* < 0.01, *** *p* < 0.001, **** *p* < 0.0001).

Neuroinflammation is another major contributor to the secondary degeneration of neurons and axons after SCI. EGCG has been reported to suppress the inflammatory cascade by inhibiting the nuclear factor kappa‐B (NF‐κB) pathway.^[^ ^56^, ^57^ ^]^ To evaluate this potential, lipopolysaccharide (LPS)‐activated BV‐2 microglia were treated with scaffold leachates and subjected to comprehensive inflammatory profiling, encompassing transcriptional regulation of key mediators, cytokine secretion dynamics, and microglial phenotypic polarization (**Figure** **5**A).

Reverse transcription–quantitative polymerase chain reaction (RT–qPCR) showed that LPS‐treated BV‐2 cells exhibited upregulated mRNA expression of interleukin‐6 (IL‐6), interleukin‐1β (IL‐1β), and inducible nitric oxide synthase (iNOS), which was significantly reduced by MPN@dECM NFs (Figure 5B). MPN@NFs also significantly reduced IL‐1β and iNOS expression. Transforming growth factor‐β1 (TGF‐β1) expression was not significantly altered by LPS stimulation but was upregulated by dECM NFs (*p* < 0.05 vs. LPS) and downregulated by both MPN@NFs and MPN@dECM NFs (*p* < 0.001). For arginase‐1 (Arg1), NFs and MPN@NFs partially reversed LPS‐induced suppression, whereas dECM NFs and MPN@dECM NFs showed no significant effect. Finally, compared to the LPS group, further suppression of lymphocyte antigen 6 complex locus C (Ly6C) expression was observed in MPN@NFs and MPN@dECM NFs (*p* < 0.0001).

Enzyme‐linked immunosorbent assay (ELISA) of cell culture supernatants revealed cytokine modulation across groups (Figure 5C). The secretion of pro‐inflammatory factors including tumor necrosis factor‐α (TNF‐α), IL‐6, and IL‐12p70 was significantly elevated after 6 and 24 h of LPS stimulation. MPN@dECM NFs showed the most significant inhibitory effect at all time points, while dECM NFs and MPN@NFs partially suppressed these factors. TGF‐β1 secretion was significantly increased after 24 h of LPS treatment, whereas both MPN@NFs and MPN@dECM NFs continuously inhibited its secretion.

Flow cytometry results showed that LPS‐activated BV‐2 cells presented a CD86^hi^/CD206^−^ classically activated pro‐inflammatory phenotype (Figure S9, Supporting Information). MPN@NFs and MPN@dECM NFs promoted a shift toward a CD86^lo^/CD206^+^ phenotype. However, in combination with RT–qPCR and ELISA data, typical M2 markers (Arg1, TGF‐β1) were not upregulated, suggesting that the process may not represent a classical M1 → M2 transition.^[^ ^58^ ^]^ The observed further downregulation of Ly6C suggests a process analogous to the Ly6C^hi^ → Ly6C^lo^ monocyte adaptive transition, which is associated with inflammation abatement and tissue repair, and attenuates fibrosis (consistent with the downregulation of the pro‐fibrotic factor TGF‐β1).^[^ ^59^, ^60^, ^61^, ^62^, ^63^ ^]^

In conclusion, MPN@dECM NFs exhibited significant inflammatory inhibitory effects, which were mainly derived from MPN NPs. The dECM also partially contributed to the anti‐inflammatory efficacy, potentially through certain anti‐inflammatory components upregulated during decellularization, such as NF‐κB inhibitor‐interacting Ras‐like protein 1 (NKIRAS1) (Figure S1D, Supporting Information). Together, MPN NPs worked synergistically with dECM to produce the enhanced comprehensive anti‐inflammatory activity of MPN@dECM NFs.

### 2.6 MPN@dECM NFs Restored Motor Function after SCI

The key to evaluating the therapeutic potential of spinal cord regenerative scaffolds is to assess the histological structural repair and functional recovery after their application to SCI animal models.^[^ ^64^, ^65^ ^]^ In this study, female C57BL/6 mice (7 weeks old) underwent T9–T10 right lateral hemisection, followed by implantation of MPN@dECM NFs, control scaffolds (NFs, dECM NFs, MPN@NFs), or no treatment (SCI), with Sham control receiving laminectomy only (Figure S10, Supporting Information).

**Figure 6:**
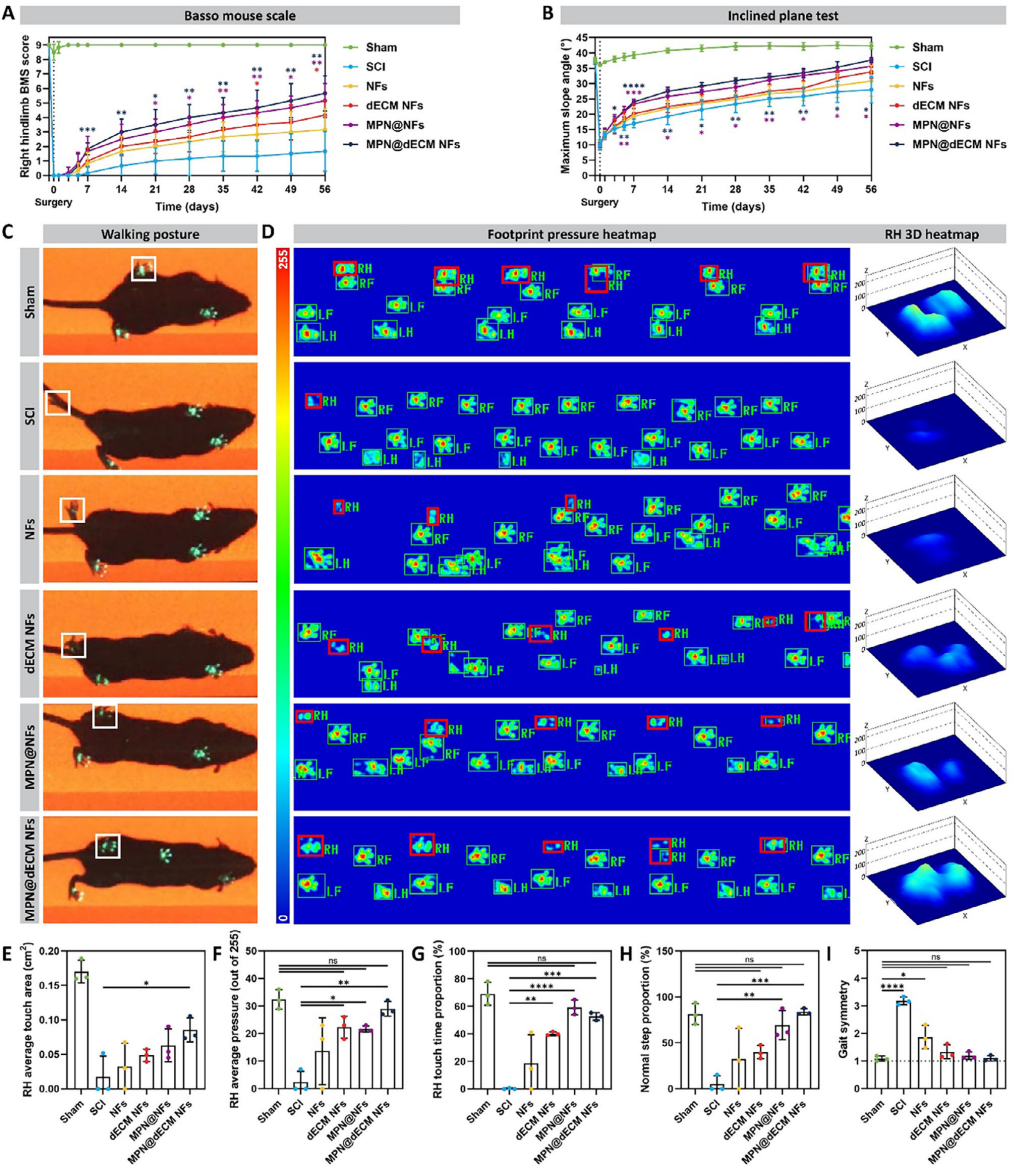
MPN@dECM NFs restored motor function after SCI. A,B) Longitudinal assessment of locomotor restoration post‐injury: BMS scores (A), and inclined plane test (B) over a period of 56 days. C–I) Gait analysis 8 weeks post‐injury: Walking posture of mice in each group (C), with white frames highlighting the right hindfeet; Footprint pressure heatmap and right hindfoot print 3D pressure heatmap (D), with red box highlighting the right hindfoot print (LF—left frontfoot; LH—left hindfoot; RF—right frontfoot; RH—right hindfoot); Quantification of right hindfoot average touch area (E), right hindfoot average pressure (F), right hindfoot touch time proportion (G), normal step proportion (H) and gait symmetry (I). Data were presented as mean ± SD (*n* = 6 (A,B), *n* = 3 (E–I)). Statistical differences were determined using two‐way ANOVA (A,B) or one‐way ANOVA (E–I) with Tukey's post hoc test (ns indicates no significance, * *p* < 0.05, ** *p* < 0.01, *** *p* < 0.001, **** *p* < 0.0001).

Restoration of motor function represents a primary objective in SCI treatment and serves as an indirect indicator of spinal cord signaling reconstruction. The Basso mouse scale (BMS) scoring was first performed to grossly evaluate the ipsilateral (right) hindlimb motor function and motor coordination of the treated mice.^[^ ^66^ ^]^ Immediately post‐surgery, all mice except the Sham group exhibited complete motor dysfunction (BMS 0) in the right hindlimb(**Figure** **6**A). At 7 days postoperatively, the MPN@dECM NFs group showed significant improvement in right hindlimb function (BMS 1.83), with limited recovery in the remaining groups. At 8 weeks postoperatively, MPN@dECM NFs showed the best functional recovery (BMS 5.67), followed by MPN@NFs (5.17) and dECM NFs (4.17, *p* < 0.05 vs. SCI 1.67), while NFs (3.17) showed no significant recovery. The inclined plate test (IPT), which evaluates the restoration of hindlimb strength in mice, again corroborated this recovery trend. While all groups experienced significant dysfunction post‐injury, the MPN@dECM NFs group demonstrated accelerated improvement in maximum slope angle, indicating rapid restoration of hindlimb strength (Figure 6B).

To further quantify multidimensional motor functional recovery, gait analysis was performed at 8 weeks postoperatively via footprint collection and motor parameter extraction.^[^ ^67^, ^68^ ^]^ The results revealed a persistent right hindlimb dragging in the SCI group (Figure 6C; Video S1, Supporting Information). The NFs group showed minimal functional restoration, characterized by dorsal/lateral paw contact dominance and negligible weight‐bearing. dECM NFs treatment resulted in a toe‐walking posture with severe axial instability, evidenced by abnormal medial displacement of the right hindpaw relative to the forepaw. MPN@NFs enabled frequent plantar stepping but intermittent coordination. In contrast, MPN@dECM NFs achieved comparable stride frequency to MPN@NFs with enhanced right hindlimb loading, improved axial stability, and consistent weight transfer (Figure 6D). Footprint pressure–time curves (Figure S11A, Supporting Information) and step‐sequence diagrams (Figure S11B, Supporting Information) further verified that the MPN@dECM NFs group closely approached Sham group in weight‐bearing capacity and motor coordination. Quantitative results further demonstrated the multidimensional benefits of MPN@dECM NFs in motor function restoration: the mean right hindfoot touch area was significantly improved, although still lower than that of Sham group; the mean right hindfoot pressure, right hindfoot touch time proportion, normal step proportion, and gait symmetry index were restored to the Sham group levels (Figure 6E–I). MPN@NFs and dECM NFs groups also exhibited partial recovery. Overall, the intergroup trend of recovery effect (MPN@dECM NFs > MPN@NFs > dECM NFs > NFs > SCI) remained consistent with BMS and IPT results, underscoring the significant benefit of MPN@dECM NFs for SCI repair.

### 2.7 MPN@dECM NFs Protected Neurons and Promoted Axonal Regeneration after SCI

To characterize the histological structural repair of the spinal cord in treated SCI mice and elucidate the histological basis for the restoration of motor function, histological analysis was performed 8 weeks postoperatively. Representative photographs of the damaged spinal cord showed that there were still significant tissue defects at the lesion sites in the SCI, NFs and dECM NFs groups, whereas the defects were completely bridged in the MPN@NFs and MPN@dECM NFs groups (Figure S12A, Supporting Information). Quantitative results showed that the MPN@NFs and MPN@dECM NFs groups exhibited lower decreases in spinal cord width at lesion sites than other groups, suggesting enhanced tissue repair and reduced peri‐lesion atrophy (Figure S12B, Supporting Information).

**Figure 7:**
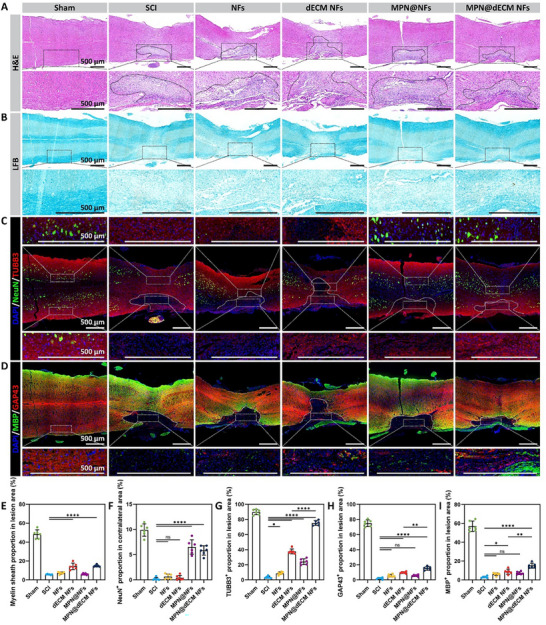
MPN@dECM NFs protected neurons and promoted axonal regeneration after SCI. A) Images of H&E staining with a black dashed curve highlighting the lesion area. Scale bar, 500 µm. B) Images of LFB staining. Scale bar, 500 µm. C) Images of dual immunofluorescence staining of NeuN and TUBB3. Scale bar, 500 µm. D) Images of dual immunofluorescence staining of GAP43 and MBP. Scale bar, 500 µm. E) Quantification of myelin sheath in LFB staining images. F–I) Quantification of NeuN (F), TUBB3 (G), GAP43 (H), and MBP (I) in immunofluorescence staining images. Data were presented as mean ± SD (*n* = 6). Statistical differences were determined using one‐way ANOVA with Tukey's post hoc test (ns indicates no significance, * *p* < 0.05, ** *p* < 0.01, **** *p* < 0.0001).

H&E staining showed that the lesion area of the MPN@dECM NFs group exhibited dense cellular structure with uniform eosinophilic protein (red), and the regenerated tissues showed distinct orientation, resembling the Sham group (**Figure** **7**A). In contrast, the intensity of eosinophilic protein staining was markedly reduced in the SCI, NFs, and MPN@NFs groups. Although dECM NFs showed abundant eosinophilic protein, the tissue were loosely organized without apparent orientation, probably due to the material's rapid degradation rate, which failed to provide long‐term structural support during regeneration.

Luxol fast blue (LFB) myelin staining demonstrated that the dECM NFs (14.3%) and MPN@dECM NFs (14.8%) groups showed significant areas of myelination at the lesion site compared to the SCI group (5.7%), whereas no discernible myelin was observed in the NFs and MPN@NFs groups (Figure 7B,E). Notably, SCI, NFs, and dECM NFs showed altered myelin distribution patterns in the contralateral side, indicating expanded demyelination accompanied by glial scarring, whereas MPN@NFs and MPN@dECM NFs confined the lesion to the injury site.

For specific detection of neurons and axons, we performed double immunofluorescence staining for neuronal nuclei antigen (NeuN) and beta‐3 tubulin (TUBB3). The results showed that the contralateral side in the SCI, NFs, and dECM NFs groups exhibited significant NeuN^+^ neuronal degeneration (< 1%, Sham group 9.9%), whereas a portion of the neurons were preserved in the MPN@NFs (6.5%) and MPN@dECM NFs (5.8%) groups (Figure 7C,F), which suggests that MPN NPs exerted a neuroprotective role and reduced secondary neuronal degeneration. On the other hand, TUBB3^+^ axons were barely detectable in the lesion area of the SCI group (3.4%), while MPN@dECM NFs group exhibited the highest density of TUBB3^+^ axons among the injury groups (75.4%), followed by dECM NFs (37.7%) and MPN@NFs (24.2%) groups (Figure 7G). Growth‐associated protein 43 (GAP43) and myelin basic protein (MBP) staining further confirmed that MPN@dECM NFs and dECM NFs significantly promoted GAP43^+^ axon sprouting and MBP^+^ myelin regeneration in the lesion area (Figure 7D,H,I). Notably, the MPN@dECM NFs group showed a superior regeneration effect to dECM NFs, and the regenerated axons were obviously oriented.

To investigate early cellular events underlying long‐term neural regeneration, we analyzed cellular proliferation and neural regeneration in cross‐sections of the caudal lesion region at 10 days post‐injury (Figure S13, Supporting Information). Immunofluorescence and EdU staining revealed robust TUBB3^+^ axon regeneration in MPN@dECM NFs, which exceeded that in other groups. All scaffolds significantly enhanced EdU^+^ cell proliferation, with MPN@dECM NFs exhibiting the highest proportion of TUBB3^+^ neurons among the proliferating cells, indicating a uniquely proneurogenic microenvironment.

In conclusion, MPN@dECM NFs demonstrated favorable neuroprotective and pro‐regenerative effects. Its neuroprotective effects are mainly derived from MPN NPs, which helped prevent lesion expansion and neuronal degeneration; whereas the axon‐regenerating capacity originated mainly from dECM and was further strengthened by the MPN NPs, which may result from the improved local microenvironment.

### 2.8 MPN@dECM NFs Modulated Microenvironment after SCI

**Figure 8:**
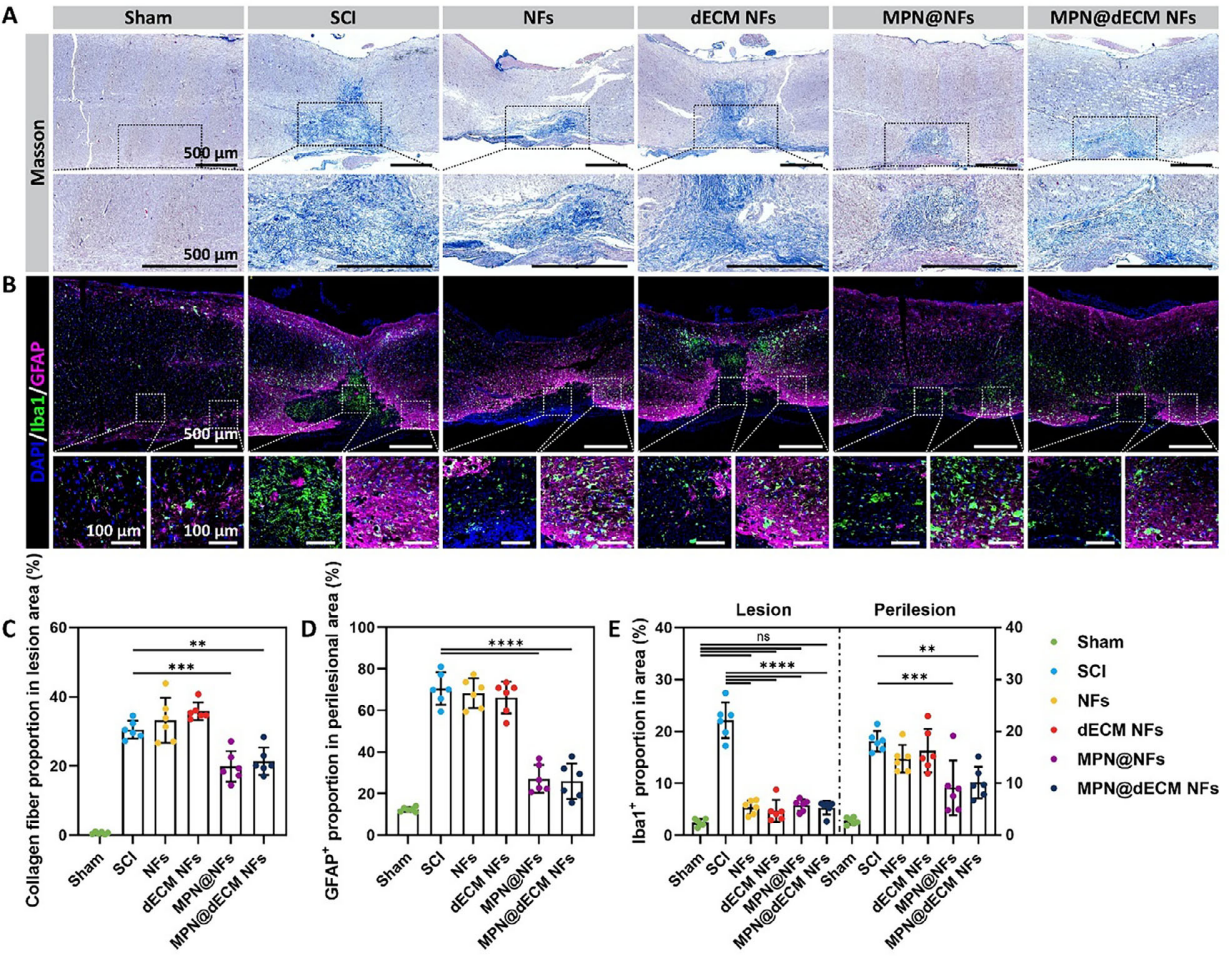
MPN@dECM NFs modulated microenvironment after SCI. A) Images of Masson's trichrome staining. Scale bar, 500 µm. B) Images of dual immunofluorescence staining of GFAP and Iba1. Scale bar, 500 µm in low‐magnification images, 100 µm in high‐magnification images. C) Quantification of collagen deposition in Masson's trichrome staining images. D,E) Quantification of GFAP (D) and Iba1 (E) in immunofluorescence staining images. Data were presented as mean ± SD (*n* = 6). Statistical differences were determined using one‐way ANOVA with Tukey's post hoc test (ns indicates no significance, ** *p* < 0.01, *** *p* < 0.001, **** *p* < 0.0001).

To elucidate the synergistic mechanism underlying the neuroprotective and axonal regenerative effects of MPN@dECM NFs, we first evaluated the acute‐phase ROS scavenging capacity of the scaffolds. Both MPN@NFs and MPN@dECM NFs significantly reduced ROS levels in the lesion area, as evidenced by ROS staining of tissue sections on day 3 and ROS measurement in tissue homogenates on day 1 post‐SCI (Figure S14, Supporting Information). We further characterized chronic‐phase scar formation at 8 weeks postoperatively by histological analysis. Masson's trichrome staining showed that MPN@NFs (19.9%) and MPN@dECM NFs (21.4%) significantly reduced fibrotic collagen deposition at the lesion site compared to the SCI group (30.5%) (**Figure** **8**A,C). Glial fibrillary acidic protein (GFAP) and ionized calcium‐binding adaptor molecule 1 (Iba1) staining showed that GFAP^+^ astrocytes were abnormally aggregated in the peri‐injury area of the SCI group (70.6%), forming dense glial scars, while MPN@NFs (27.2%) and MPN@dECM NFs (25.9%) significantly inhibited scar formation. Moreover, Iba1^+^ microglia/macrophage infiltration persisted in both the lesion area and peri‐lesion area during the chronic phase in the SCI group, whereas MPN@NFs and MPN@dECM NFs showed significant suppression (Figure 8B,D,E). These results demonstrate that MPN@dECM NFs effectively mitigated oxidative stress in the acute phase, which subsequently contributed to reduced chronic inflammation and scar formation. This comprehensive amelioration of the pathological microenvironment underscores not only its superior axonal regenerative capacity but also its distinct neuroprotective advantage over dECM NFs alone, ultimately promoting neural repair and functional recovery.

Based on integrated cellular and animal evidence, we demonstrated that MPN@dECM NFs protected spared neurons from secondary degeneration via attenuating excitotoxicity and suppressing microglia‐mediated neuroinflammation, while promoting a permissive microenvironment for axonal regeneration and remyelination, consequently facilitating the restoration of motor function. These results align with our therapeutic objectives.

Neuroprotection and axonal regeneration constitute dual imperatives for effective spinal cord repair. Current biomaterial strategies primarily promote axonal regrowth through topographical guidance or regenerative signaling molecules, as exemplified by dECM scaffolds that deliver pro‐regenerative cues.^[^ ^10^, ^11^, ^14^ ^]^ However, structural reconnection supported solely by pro‐regenerative signals is insufficient. The persistently hostile injury microenvironment perpetuates degenerative cascades, where the loss of spared neurons, demyelination, and degeneration of nascent axons further impair signal transmission, creating self‐perpetuating barriers to functional restoration. Critically, spared neurons serve as indispensable neural relays, maintaining residual circuitry integrity and facilitating signal transmission.^[^ ^69^, ^70^, ^71^ ^]^ Their preservation represents a foundational prerequisite for meaningful functional outcomes, yet remains inadequately addressed in current clinical approaches and scaffold design paradigms. For instance, clinical methylprednisolone pulse therapy offers transient edema suppression but fail to halt neurodegeneration while incurring significant complications.^[^ ^72^ ^]^

In the present study, MPN@dECM NFs scaffold bridged this therapeutic gap through dual‐pathway integration. By embedding pH‐responsive Mg–EGCG MPN NPs within aligned dECM fibers, we achieved spatiotemporally coordinated bioactivity: sustained release of Mg^2+^ blocked excitotoxic Ca^2+^ influx, while EGCG modulated microglial responses and astrocyte activation. This neuroprotective axis robustly preserved neuronal viability during the critical 8‐week degenerative phase, concurrently attenuating glial scarring and promoting a regeneration‐permissive microenvironment (Figures 7 and 8). Consequently, dECM's innate regenerative capacity was fully unleashed, yielding enhanced axonal regrowth and remyelination. Notably, motor function assessments confirmed superior functional recovery in neuroprotective scaffold groups (MPN@NFs and MPN@dECM NFs) versus non‐MPN groups (Figure 6). Specifically, MPN@NFs group achieved significantly better functional outcomes despite exhibiting less axonal regeneration than dECM NFs group, demonstrating that neuroprotection functionally compensates for regenerative deficits. This evidence establishes neuronal preservation as an essential prerequisite for effective repair.

Collectively, this study developed a dual‐functional regenerative scaffold that synergistically addresses neuroprotection and axonal regeneration, establishing a mechanistically advanced paradigm in scaffold design for SCI treatment.

## 3 Conclusion

In this study, we designed a spinal cord regenerative scaffold based on the characteristics of the pathological process of SCI. First, dECM was derived from decellularized porcine spinal cord, while Mg–EGCG MPN NPs were synthesized through MPN self‐assembly. These two components were then integrated through co‐electrospinning to form an oriented fibrous scaffold, MPN@dECM NFs, which recapitulates the architecture of native spinal cord ECM. This scaffold exhibited well‐aligned fibers with homogeneous diameter distribution, appropriate porosity, excellent hydrophilicity, appropriate degradation kinetics, and effective radical scavenging capacity. At the cellular level, MPN@dECM NFs inhibited excitotoxic neuronal death by suppressing Ca^2+^ influx, alleviating oxidative damage and maintaining mitochondrial homeostasis, and suppressed neuroinflammatory responses by downregulating pro‐inflammatory gene expression and pro‐inflammatory factor secretion in activated microglia. In a mouse SCI model, MPN@dECM NFs showed favorable microenvironmental modulation in the chronic phase, protected spared neurons from secondary degeneration, and directed axonal regeneration, consequently promoting the restoration of motor function in SCI mice. In summary, the composition and structural design of MPN@dECM NFs confer specific antagonism against excitotoxicity and neuroinflammation, effectively enhancing neuroprotection and axonal regeneration after SCI, thereby offering a promising strategy for achieving functional recovery in SCI treatment.

## 4 Experimental Section

The detailed methods can be found in the Supporting Information.

## Conflict of Interest

The authors declare no conflict of interest.

## Supporting information



Supporting Information

Supplemental Video 1

## Data Availability

The data that support the findings of this study are available from the corresponding author upon reasonable request.
