# Supplementary material for: Neuroprotection and Axonal Regeneration via ECM‐Mimetic Nanofibers Incorporating Metal–Phenolic Network Nanoparticles Toward Spinal Cord Injury Repair
Source: Adv Sci (Weinh). 2025 Oct 27;13(1):e13825. doi: 10.1002/advs.202513825 (PMC12767005; doi:10.1002/advs.202513825)
Supplement: Supplementary file 1 — Supporting Information [file ADVS-13-e13825-s002.docx]

Supporting Information

Neuroprotection and Axonal Regeneration via ECM-Mimetic Nanofibers Incorporating Metal–Phenolic Network Nanoparticles toward Spinal Cord Injury Repair

*Shu Chen, Bixue Wang, Qiya Zhang, Changsheng Liu*, and Xi Chen**

Key Laboratory for Ultrafine Materials of Ministry of Education, Frontiers Science Center for Materiobiology and Dynamic Chemistry, Engineering Research Center of Biomedical Materials of Ministry of Education, School of Materials Science and Engineering, East China University of Science and Technology, Shanghai 200237, China

E-mail: chenxi@ecust.edu.cn

1. Supplemental Figures


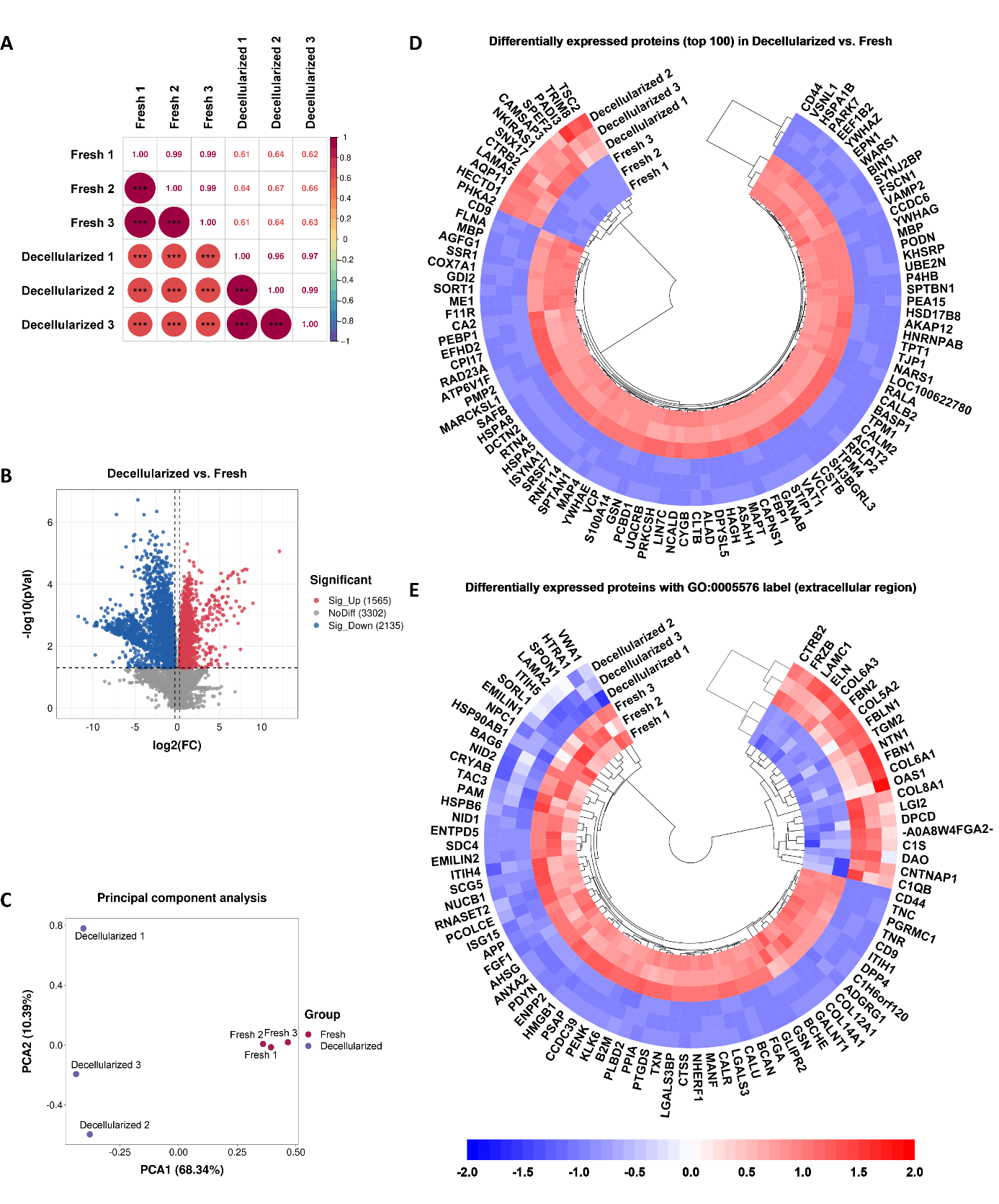


**Figure S1.** Proteomic analysis of dECM. A) Pearson correlation coefficient heatmap of fresh and decellularized spinal cord. B) Volcano plot of differentially expressed proteins between fresh and decellularized spinal cord tissues. Red dots represent significantly upregulated proteins (decellularized vs. fresh), blue dots denote significantly downregulated proteins, and gray dots indicate proteins with no significant differential expression (t-test, |log2FC| > 0.263 and *p* < 0.05). The dashed line corresponds to the significance threshold. C) PCA projection (prcomp implementation) of fresh and decellularized spinal cord protein profiles after Z-score standardization. D,E) Heatmap clustering of top 100 (D) or extracellular region-associated (GO:0005576) (E) differentially expressed proteins between decellularized and fresh spinal cord. Protein contents were presented using Z-score.


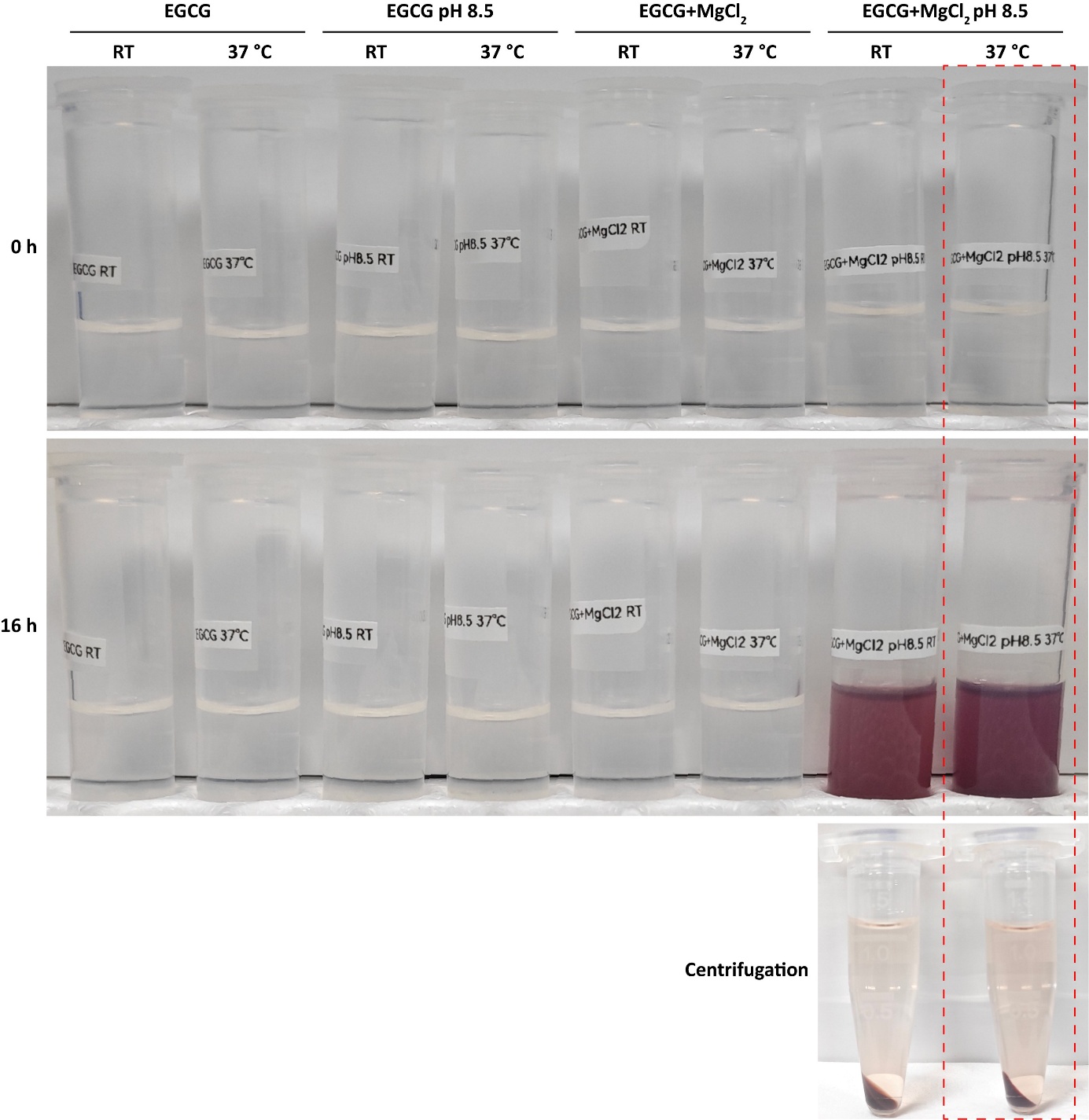


**Figure S2.** Changes in EGCG and EGCG/MgCl_2_ in ethanol solutions at different pH and temperatures. The red box indicates the synthesis condition for MPN NPs in this study.


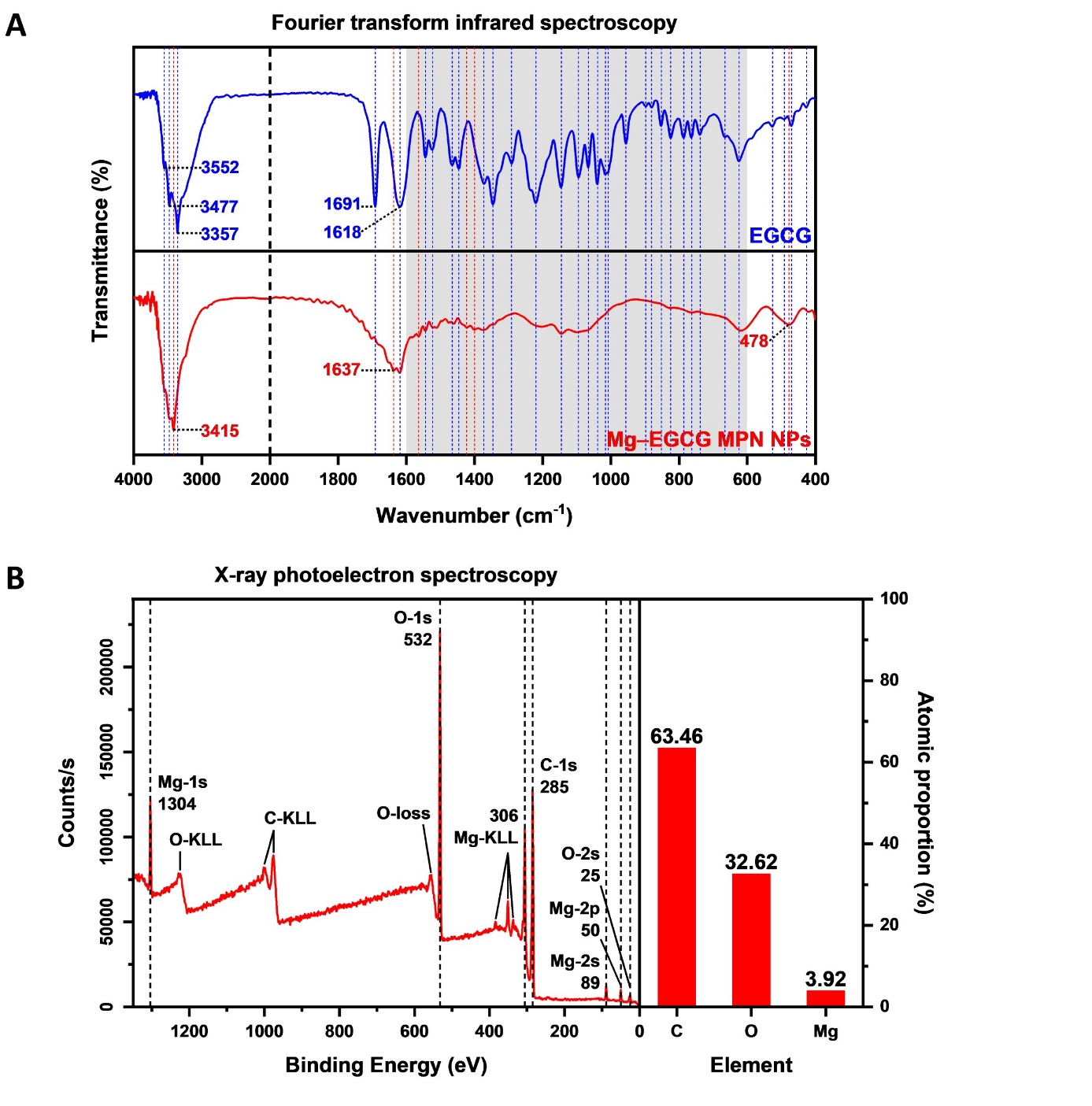


**Figure S3.** A) FTIR spectra (4000–400 cm^−1^) comparison of EGCG molecules and Mg–EGCG MPN NPs. B) XPS survey (1350–0 eV) of Mg–EGCG MPN NPs.


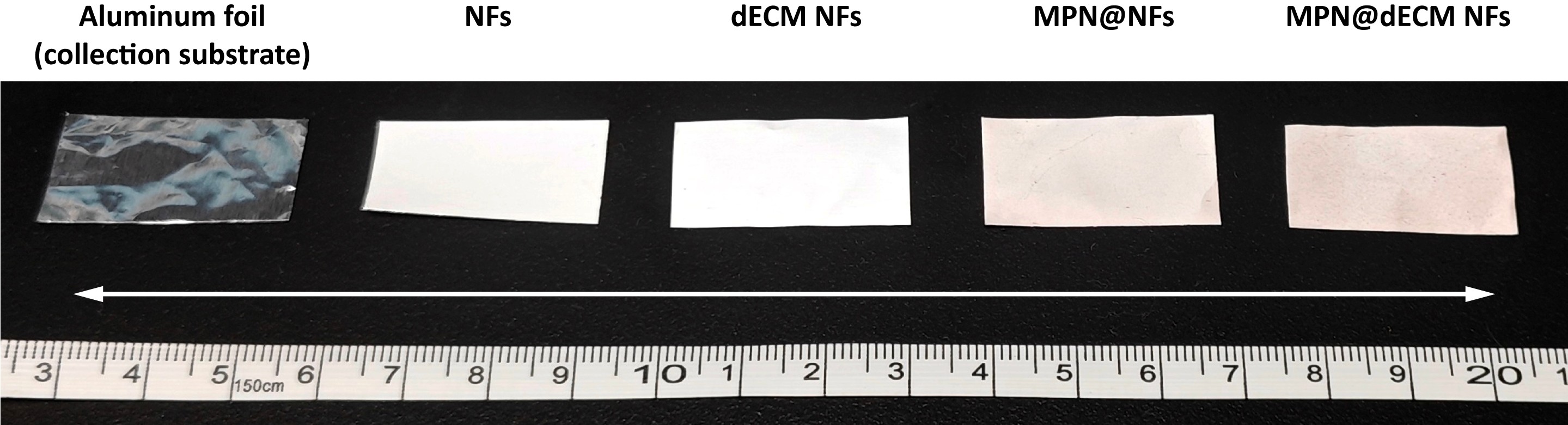


**Figure S4.** Macroscopic photographs of electrospun fiber membranes. The white double-headed arrow indicates the fiber direction.


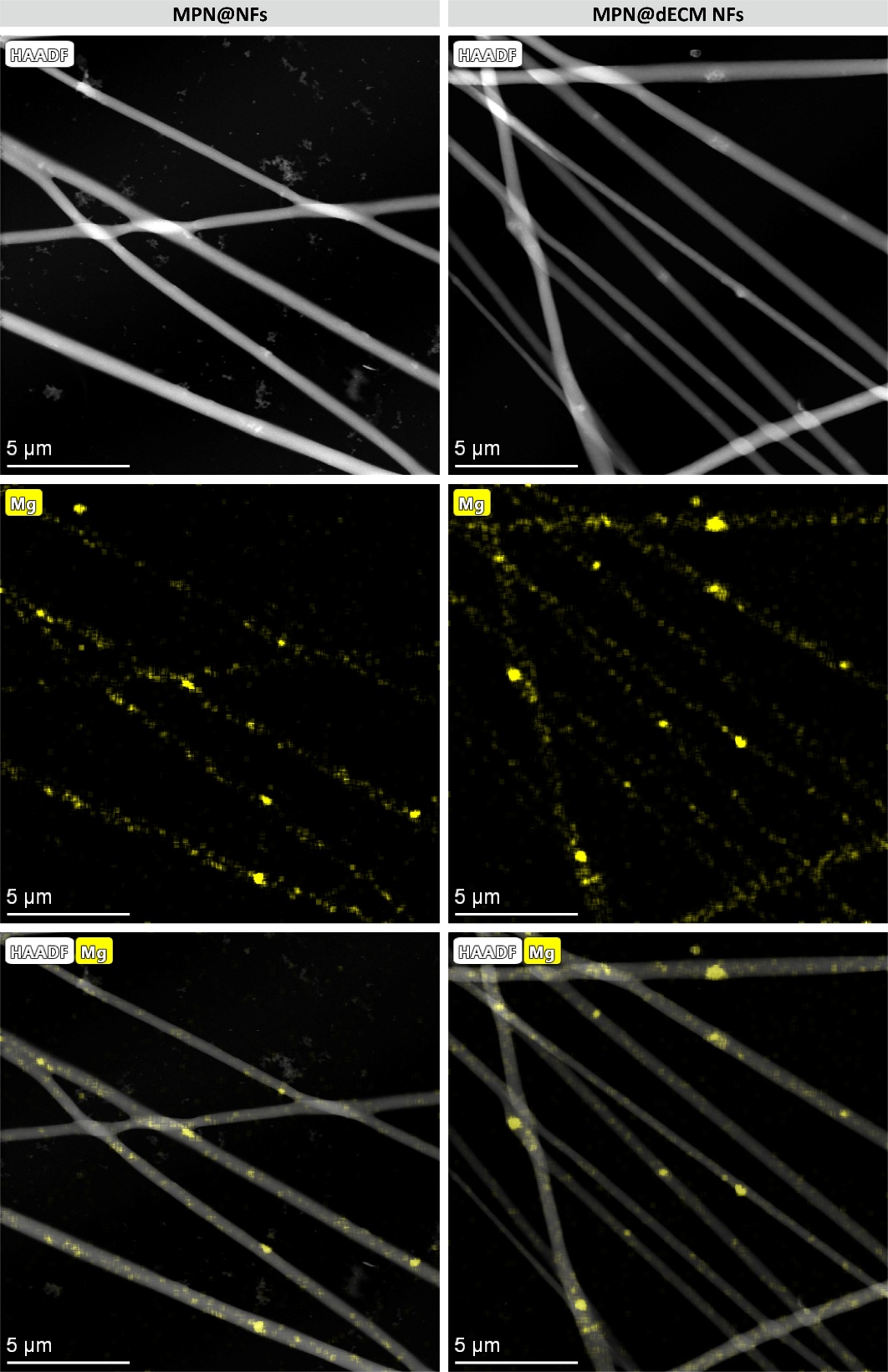


**Figure S5.** Mg-mapping images of MPN@NFs and MPN@dECM NFs by TEM-EDS. Scale bar, 5 μm.


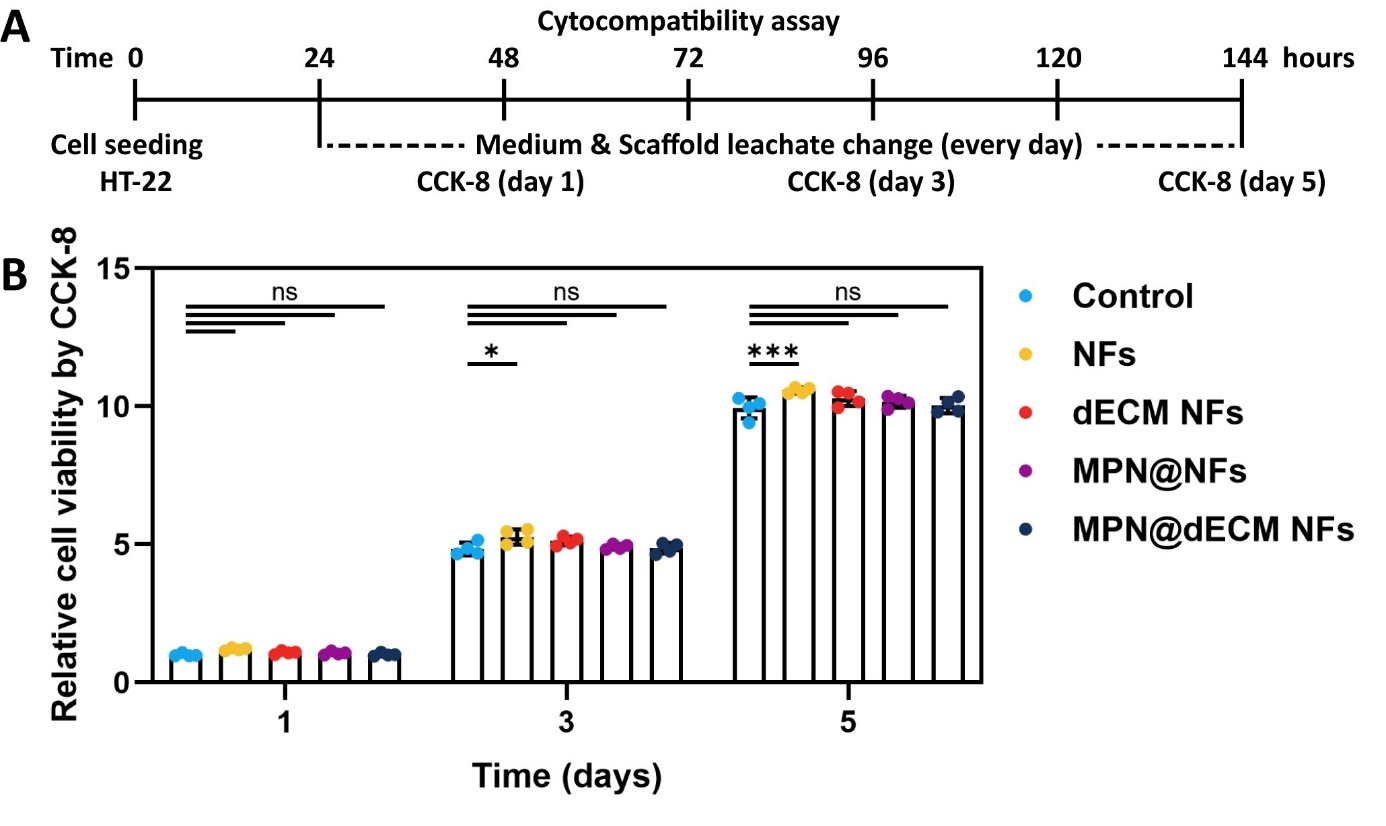


**Figure S6.** Cytocompatibility assay of MPN@dECM NFs. A) Experimental workflow. B) Relative cell viability assessed by CCK-8 assay. Data were presented as mean ± SD (*n* = 4). Statistical differences were determined using two-way ANOVA with Tukey’s post hoc test (ns indicates no significance, * *p* < 0.05, *** *p* < 0.001).


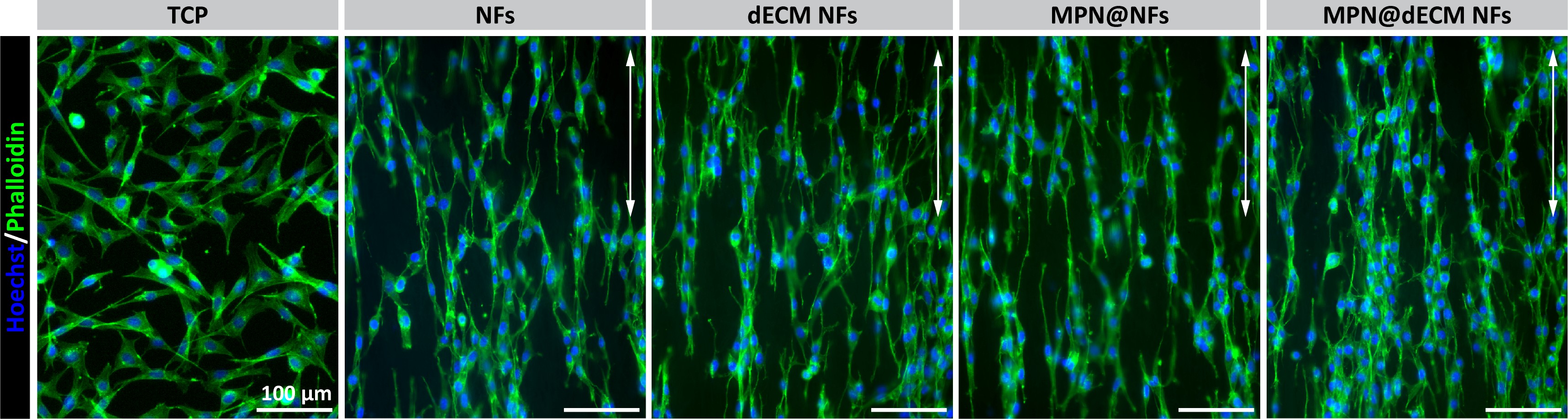


**Figure S7.** Cytoskeletal staining images of HT-22 cells seeded on fiber membranes. White double-headed arrows indicate the direction of cell extension, which aligns with fiber orientation. Scale bar, 100 μm. TCP, tissue culture plate.


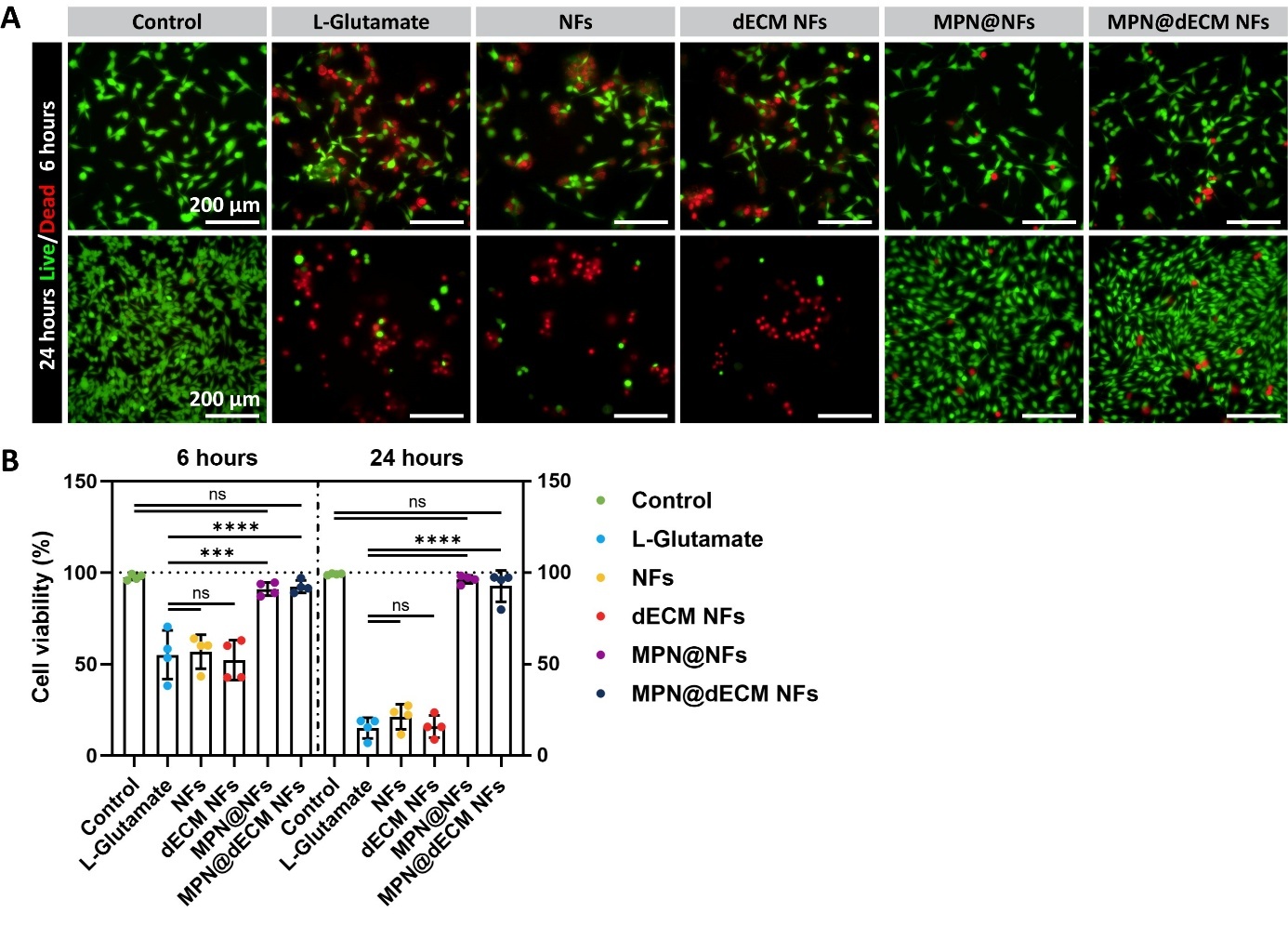


**Figure S8.** A) Live/dead cell staining of in vitro excitotoxicity inhibition assay (Calcein-AM/PI). Scale bar, 200 μm. B) Quantitative result of live/dead cell staining. Data were presented as mean ± SD (*n* = 4). Statistical differences were determined using one-way ANOVA with Tukey’s post hoc test (ns indicates no significance, *** *p* < 0.001, **** *p* < 0.0001).


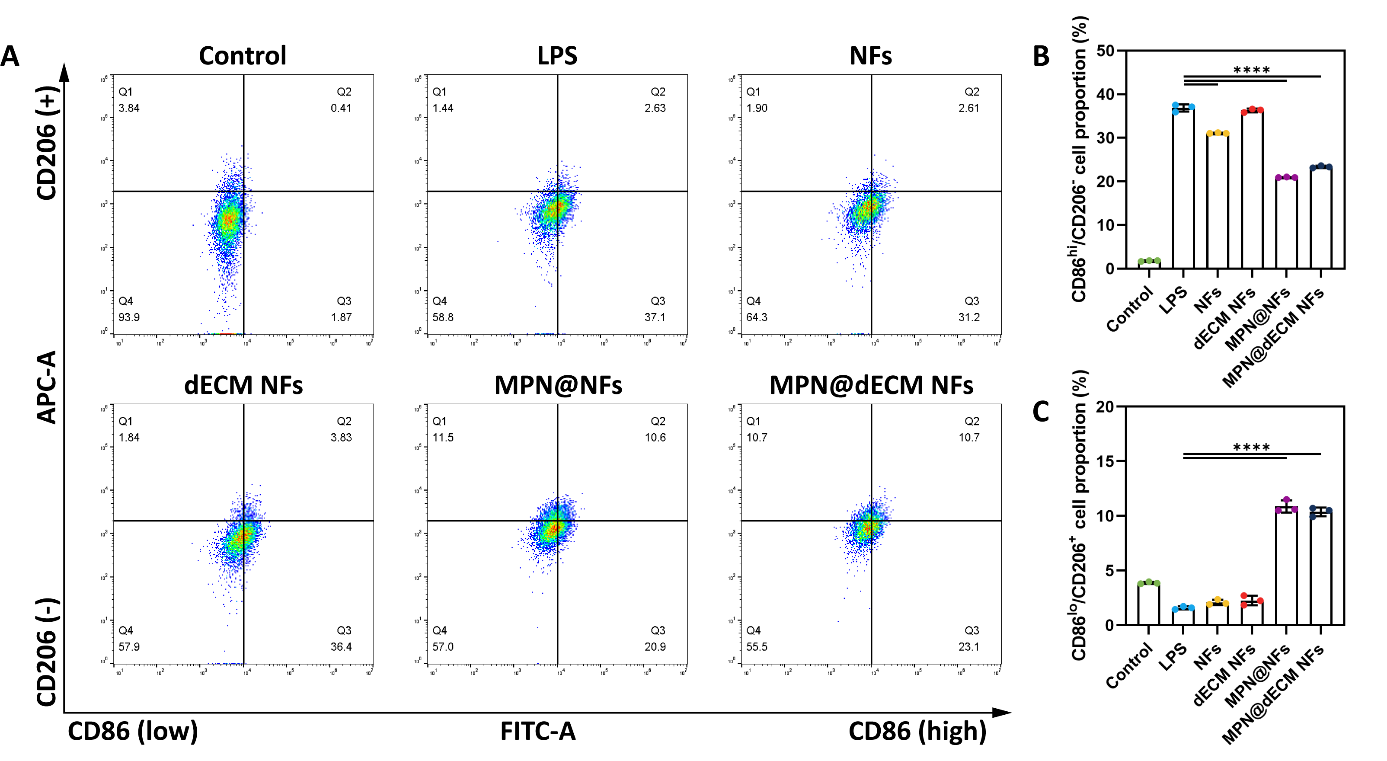


**Figure S9.** A) Flow cytometry analysis of in vitro immunomodulation assay (CD86/CD206). B,C) Qualification of CD86^hi^/CD206^−^ (B) and CD86^lo^/CD206^+^ (C) cell proportion. Data were presented as mean ± SD (*n* = 3). Statistical differences were determined using one-way ANOVA with Tukey’s post hoc test (**** *p* < 0.0001).


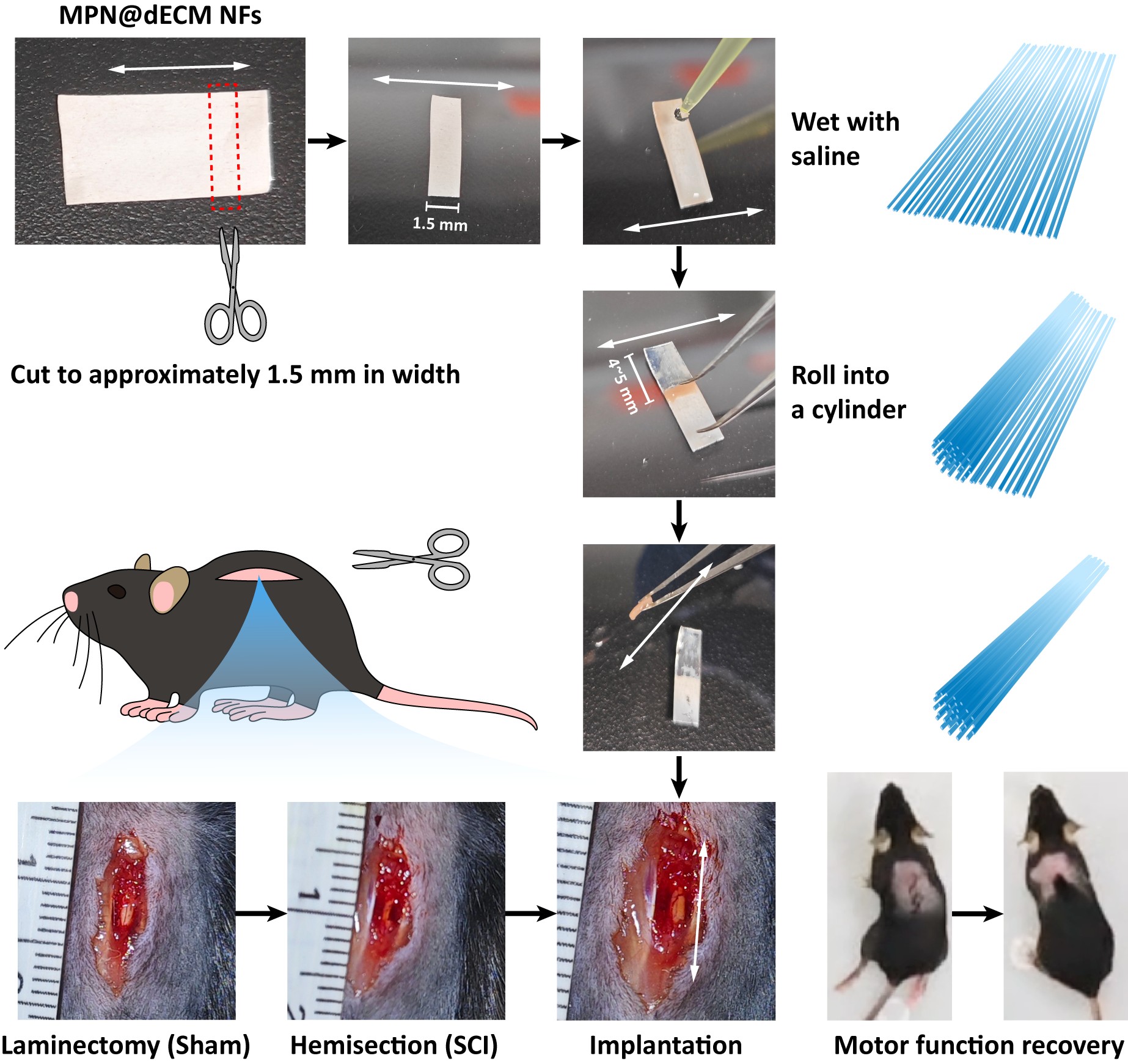


**Figure S10.** Schematic diagram of spinal cord injury modeling and scaffold implantation procedure. The white double-headed arrow indicates the fiber direction.


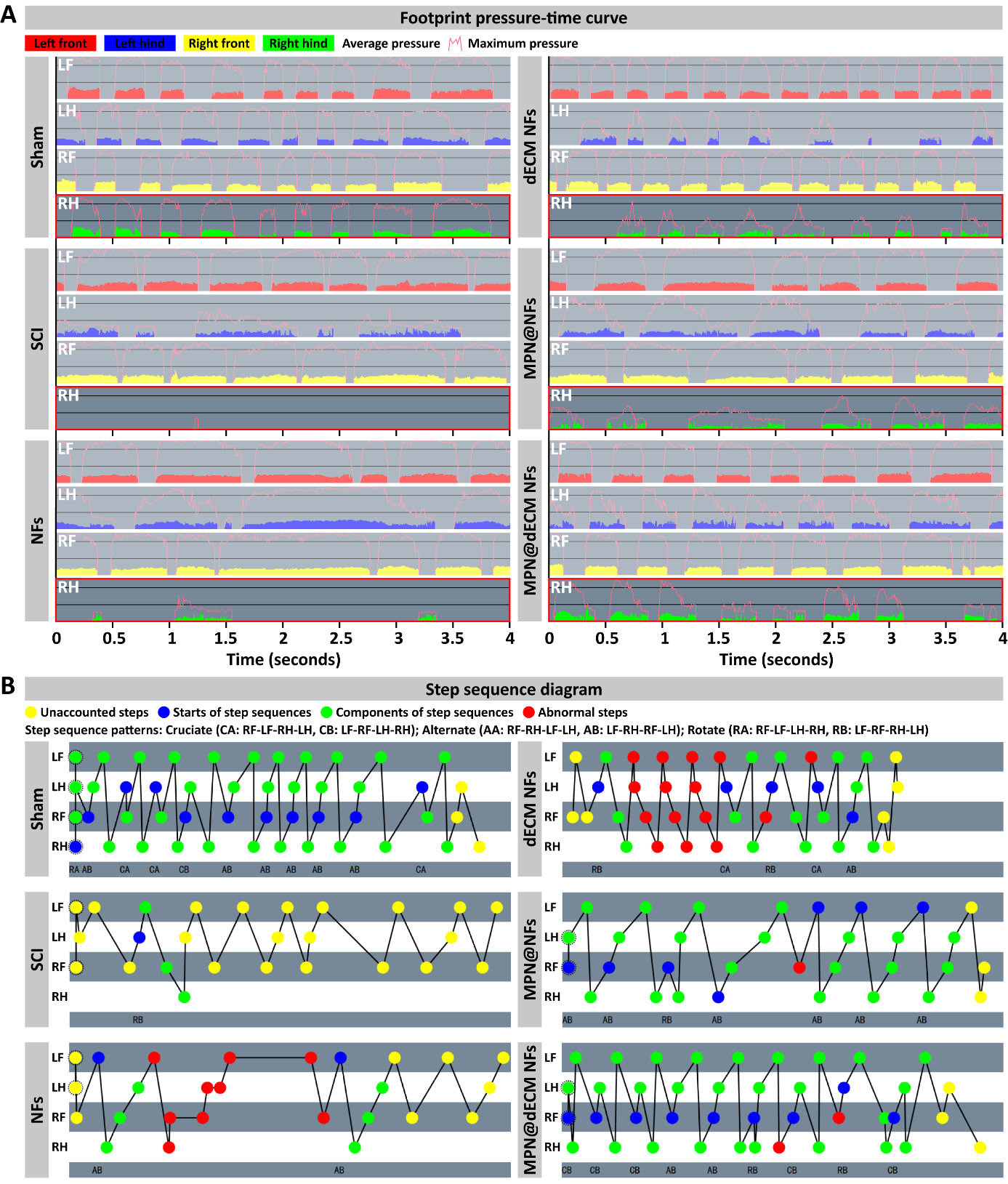


**Figure S11.** Supplemental data for gait analysis 8 weeks post-injury. A) Footprint pressure-time curve, with the curves of the right hindfeet highlighted. B) Step sequence diagram with different colors marking starts of step sequences, components of step sequences, abnormal steps and unaccounted steps.


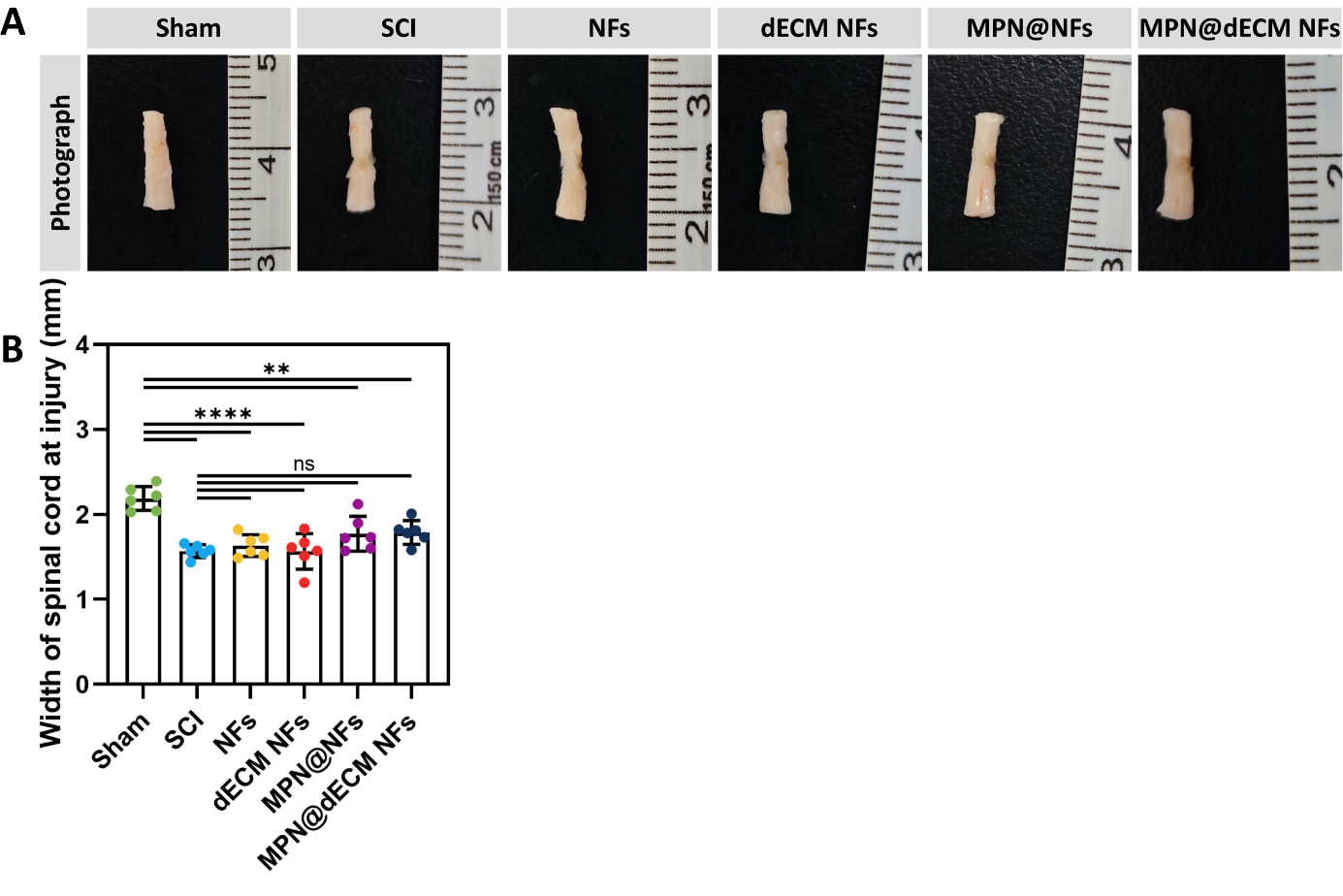


**Figure S12.** A) Photographs of injured spinal cord (8 weeks). B) Quantification of spinal cord width at the lesion site. Data were presented as mean ± SD (*n* = 6). Statistical differences were determined using one-way ANOVA with Tukey’s post hoc test (ns indicates no significance, ** *p* < 0.01, **** *p* < 0.0001).


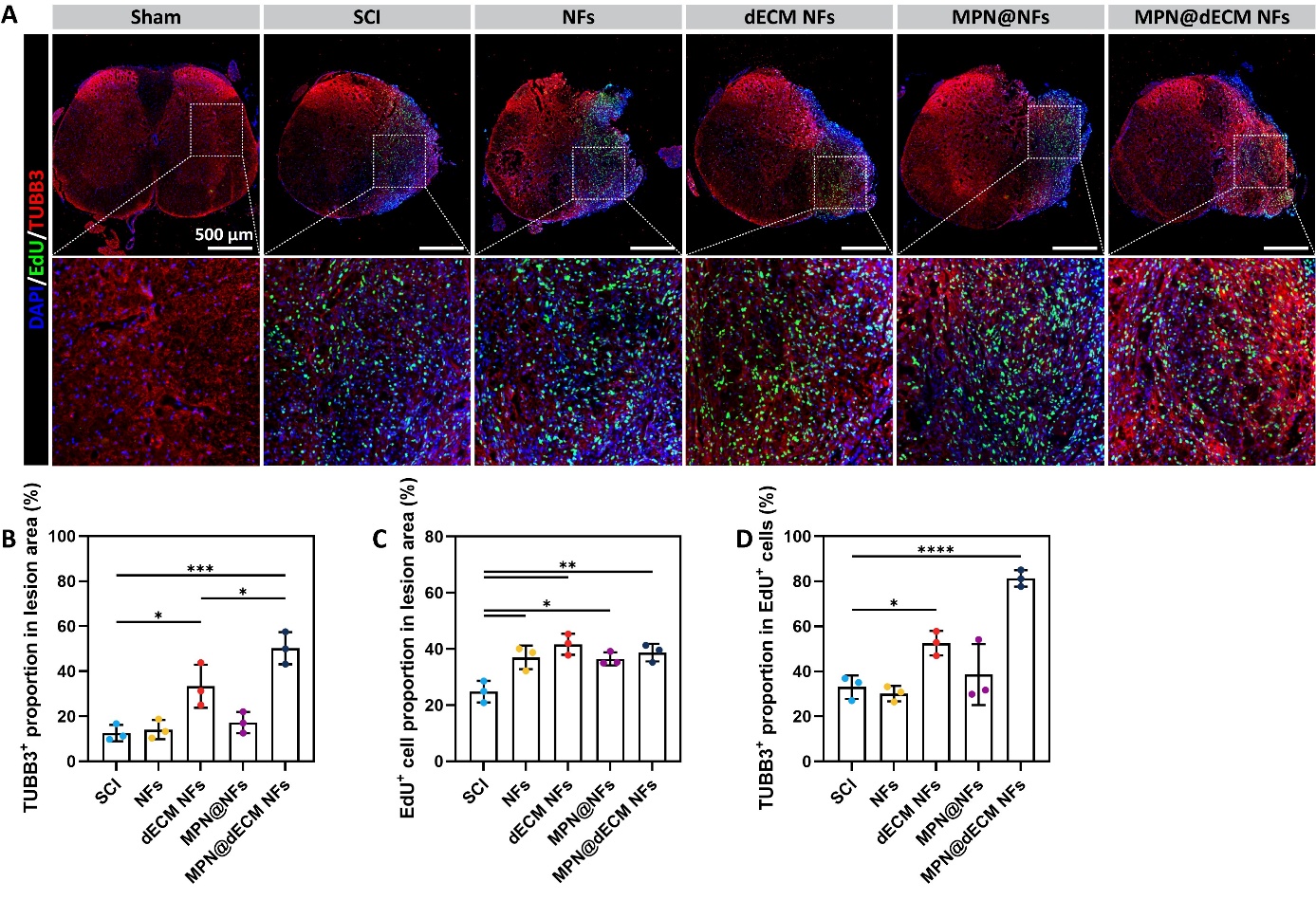


**Figure S13.** MPN@dECM NFs promote neural regeneration in the caudal region of the injury site on day 10 post-surgery. A) Representative cross-sectional images of the caudal injury region, showing EdU and immunofluorescence staining for TUBB3. Scale bar, 500 μm. B–D) Quantitative analysis of TUBB3 (B), EdU (C), and co-localization (D). Data were presented as mean ± SD (n = 3). Statistical differences were determined using one-way ANOVA with Tukey’s post hoc test (* p < 0.05, ** p < 0.01, *** p < 0.001, **** p < 0.0001).


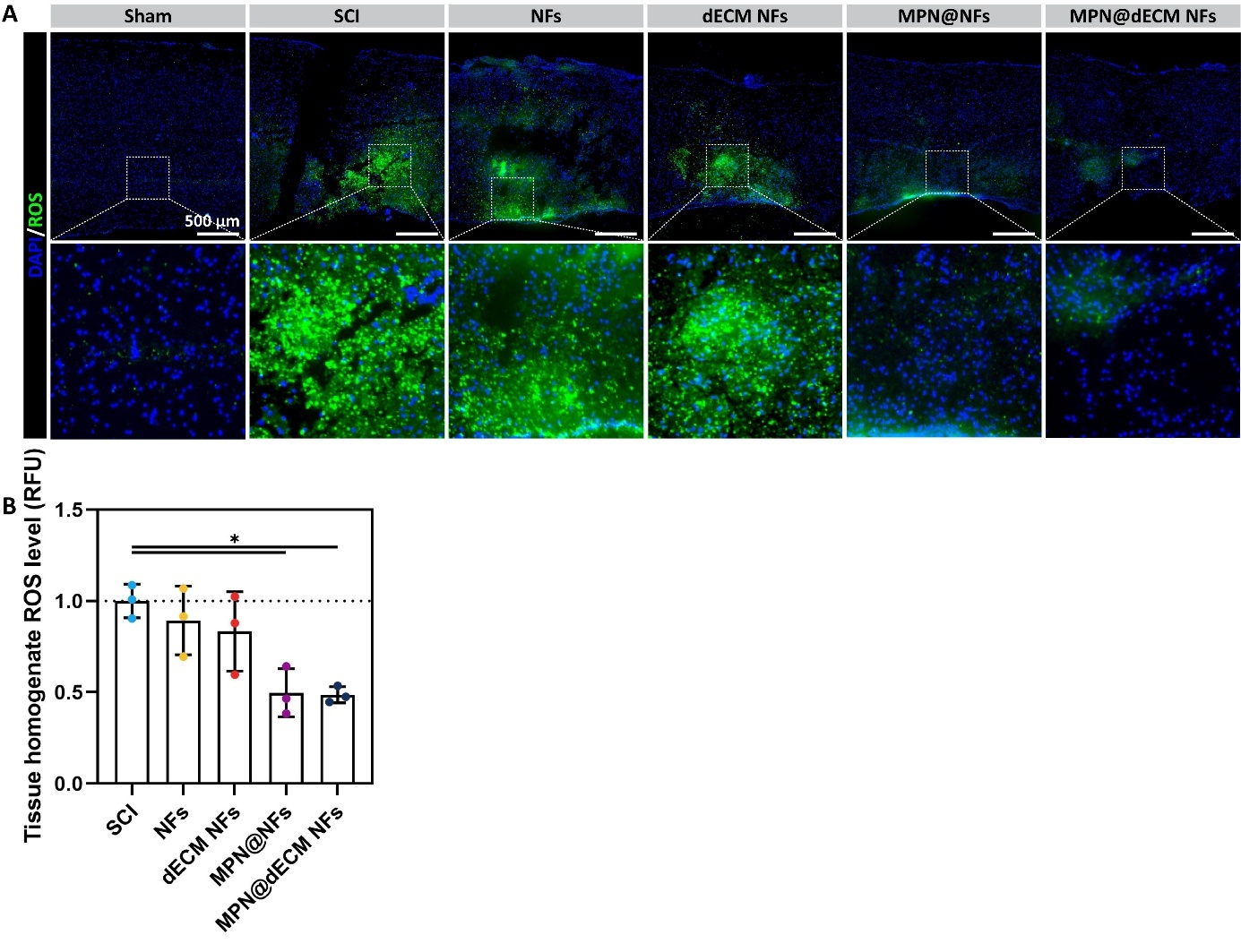


**Figure S14.** MPN@dECM NFs scavenge acute-phase ROS at the SCI lesion site. A) Representative images of ROS staining in spinal cord tissues on day 3 after surgery. Scale bar, 500 μm. B) ROS levels measured in tissue homogenates on day 1 post-surgery. Data were presented as mean ± SD (n = 3). Statistical differences were determined using one-way ANOVA with Tukey’s post hoc test (* p < 0.05).

2. Supplemental Video

Supplemental Video 1 shows the walking posture and real-time recorded footprints of treated SCI mice.

3. Experimental Section

*Preparation of dECM*: Fresh porcine spinal cord was removed from the dura mater and arachnoid, retaining the pia mater, and subsequently frozen at −20 °C. Next, the spinal cord was rapidly cut cross-sectional into 0.5-cm segments in the frozen state. The spinal cord segments were placed vertically in a petri dish, fully restored to room temperature, and then frozen at −20 °C for 1 h. The freeze–thaw cycle was repeated until the spinal cord segments returned to room temperature for the third time. Subsequently, spinal cord segments were placed in 50 mL conical flasks at 15 segments per flask, 25 mL of ultrapure (UP) water was added, and shaken at 120 rpm for 6 h at 37 °C on a constant temperature shaker. Then, maintaining 37 °C and 120 rpm, the following wash solutions were exchanged in sequence, 25 mL each: Triton X-100 (#T8787, Sigma-Aldrich) (3% v/v in UP water, 6 h), UP water (20 min × 3), sodium deoxycholate (SDC, #30970, Sigma-Aldrich) (4% w/v in UP water, 10 h), UP water (20 min × 3), DNase I (#HY-108882, MedChemExpress) (40 Kunitz U/mL with 1 mol/L NaCl/0.015 mol/L MgCl_2_/0.001 mol/L CaCl_2_ in UP water, 4 h), UP water (20 min × 3). The above washing process was repeated 3 times in total. Next, 4% (v/v in UP water) ethanol (37 °C, 120 rpm, 4 h) and UP water (37 °C, 120 rpm, 1 h × 3) were used sequentially to remove the residual detergent. The decellularized spinal cord segments were fully homogenized by wet grinding at 4 °C and then filtered using a 40-μm cell strainer to remove residual large particles. The homogenate was lyophilized to obtain dECM fine powder, which was then stored dry at −20 °C.

*Characterization of dECM*: To confirm the effectiveness of the decellularization method, H&E staining, DAPI staining, DNA quantification, sGAG quantification and collagen quantification were performed. For H&E staining, decellularized spinal cord segments were fixed using 4% paraformaldehyde (PFA), followed by ethanol dehydration, xylene clearing, and paraffin embedding, then cut into 4-μm slices using a microtome (RM2016, Leica). A commercial H&E staining kit (#G1005, Servicebio, China) was used. Briefly, according to the manual, the slices were dewaxed and rehydrated, then sequentially immersed in hematoxylin, acid-ethanol, ammonia, and eosin, and finally dehydrated, cleared, and mounted with neutral balsam. The slices were then observed and photographed using a slice scanner (Pannoramic MIDI, 3DHISTECH, Hungary), and the images were processed using the dedicated software (CaseViewer). For DAPI staining, after PFA fixation, decellularized spinal cord segments were dehydrated using concentrated sucrose solution, then immersed in the optimal cutting temperature (OCT) compound (#4583, Sakura Finetek), snap-frozen in liquid nitrogen, and cut into 14-μm slices with a cryotome (Minux FS800, RWD, China). The slices were soaked in water and then mounted with the anti-fading mounting medium with DAPI (Servicebio), observed and photographed using a slice scanner (APX100, Olympus, Japan), and the images were processed using the dedicated software (OlyVIA). For DNA quantification, total DNA was extracted using the DNeasy Blood & Tissue Kit (#69504, QIAGEN, Germany). The concentration of double-stranded DNA was then detected using a microvolume spectrophotometer (NanoDrop OneC, Thermo Fisher) and the DNA content was calculated accordingly. Quantification of sGAG was performed by extracting sGAG using the Tissue sGAG Quantification Assay Kit (#GMS19239.2, Genmed, USA) and visualizing the color, followed by detection of absorbance using a microplate reader (SpectraMax Plus 384, Molecular Devices, USA) and calculation of the content. The collagen content was converted from the hydroxyproline (HYP) content by the ratio collagen content = HYP content × 100/13. HYP was quantified by extracting HYP using the HYP Content Assay Kit (#BC0250, Solarbio, China) and visualizing the color, followed by detection of absorbance with the microplate reader and calculation of the content.

*Proteomic analysis*: Proteomic analysis was used to detect changes in protein composition after decellularization. The experimental procedures for proteomic analysis were conducted as follows. Samples were ice-bathed for 5 min and homogenized in a 2 mL centrifuge tube containing steel spheres and lysis buffer (8 mol/L urea, 50 mmol/L Tris–HCl) with cOmplete Protease Inhibitor Cocktail (#11836170001, Roche, Switzerland), then centrifuged (20 000 × g, 4 °C, 15 min) and the supernatant collected. Reduction and alkylation were achieved by adding 10 mmol/L dithiothreitol (DTT, #ST040, Beyotime, China) (37 °C, 1 h) and 20 mmol/L iodoacetamide (IAA, #I105563, Aladdin, China) (dark conditions, 30 min), respectively. Protein quantification was performed by the Bradford method: gradient concentrations of bovine serum albumin (BSA, #P0007, Beyotime) were prepared and mixed with Coomassie Brilliant Blue G250 (#18.001.05, Affinibody, Switzerland), and the absorbance (595 nm) was detected in order to develop a standard curve, then the sample proteins were detected and the concentrations were calculated from the standard curve. For digestion, 150 μg proteins were subjected to tryptic cleavage (1:50 enzyme ratio, 37 °C, 14–16 h), with subsequent peptide desalination via solid phase extraction (SPE) cartridges (#186000383, Waters, USA) and lyophilization. Nano-liquid chromatography–tandem mass spectrometry (Nano-LC–MS/MS) analysis utilized a Vanquish Neo UHPLC system (Thermo Fisher) with ES906 (Thermo Fisher) column (150 mm) employing an 8-min acetonitrile gradient (4–99% mobile phase B) at 2.5 μL/min flow rate, coupled to an Astral mass spectrometer (Thermo Fisher) operating in data independent acquisition (DIA) mode (380–980 m/z, 240 000 resolution, 25% collision energy). DIA-NN software performed library-free data processing against UniProt sequences using Trypsin/P specificity (≤ 2 missed cleavages) with carbamidomethylation as fixed modification and oxidation/acetylation as variable modifications, applying 1% false discovery rate (FDR) filtering for reliable protein identification and quantification.

Proteomic data processing and statistical analyses were conducted in R (v4.0.0). Raw protein abundances were median normalized. Principal component analysis (PCA) was performed with prcomp algorithm on Z-score standardized data to evaluate global proteomic variations. Pairwise correlations between samples were calculated using Pearson correlation coefficients (R cor package). Differential protein expression was identified by Student’s t-test (*p* < 0.05) with a threshold of |log2FC| > 0.263. Gene Ontology (GO) annotation was applied to classify biological functions of the differentially expressed proteins. Hierarchical clustering heatmaps of differentially expressed proteins (|log2FC| > 0.263, *p* < 0.05) were generated in Origin software (v10.1.5.132, OriginLab, USA) using Z-score normalized values, with clustering based on Euclidean distance and group average cluster method.

*Synthesis of MPN NPs*: First, EGCG (#E808891, Macklin, China) solution (4 mmol/L in ethanol) and MgCl_2_ (#M813765, Macklin) solution (80 mmol/L in ethanol) were prepared separately. Next, the two solutions were mixed in equal volumes of 10 mL each in a 50-mL centrifuge tube. Then, 1.5 mL of NaOH solution (0.1 mol/L in UP water) was added dropwise to adjust the pH to about 8.5. The mixed solution was subsequently incubated on a thermal mixer at 37 °C, 800 rpm for 16 h. The obtained brown mixture was ultrasonicated for 5 min to disperse the NPs and then centrifuged at 16 000 × g for 10 min. The supernatant was removed, appropriate amount of ethanol was added and the mixture was ultrasonicated for 5 min to wash the NPs and centrifuged again. After repeating the washing 2 times, appropriate amount of UP water was added and the mixture was ultrasonicated for 5–10 min to completely disperse the NPs. Finally, the mixture was snap-frozen by liquid nitrogen and then lyophilized to obtain dry MPN NPs, which were then stored dry and protected from light at −20 °C.

*Characterization of MPN NPs*: The morphology, chemical composition, and release profile of Mg–EGCG MPN NPs were systematically characterized. TEM imaging was performed on purified NPs ultrasonically dispersed in ethanol (5–10 min) and deposited onto carbon-coated copper grids using Talos F200X (Thermo Fisher). EDS elemental mapping of C (Kα), O (Kα), and Mg (Kα) was conducted in high-angle annular dark-field scanning transmission electron microscopy (HAADF-STEM) mode, and mapping images were merged in Adobe Photoshop (v20.0.0). DLS analysis was carried out on NPs dispersed in ultrapure water (5–10 min sonication) to measure hydrodynamic diameter and zeta potential using Zetasizer Nano series (Malvern, UK). FTIR spectra (4000–400 cm^−1^) were recorded from dried NPs pressed into potassium bromide (KBr) pellets using INVENIO S spectrometer (Bruker, Germany). XPS survey scans (1350–0 eV) were acquired with ESCALAB 250Xi system (Thermo Fisher). For Mg^2+^ release kinetics, NPs (10 mg) were incubated in pH-adjusted saline solutions (4.5/6.0/7.4, adjusted with 0.1 mol/L HCl/NaOH, 50 mL) at 37 °C with 300 rpm shaking. Aliquots (500 μL) collected at 3, 6, 12, 24, and 48 h were centrifuged (16 000 × g, 4 °C, 10 min), and supernatants (200 μL) were diluted 50-fold with dilute HCl for Mg^2+^ quantification via Agilent 5800 ICP-OES (USA). Cumulative release percentages were calculated based on the total Mg content determined by XPS analysis, with the baseline release defined as 0% at 0 h.

*Fabrication of MPN@dECM NFs*: All fibrous scaffolds were fabricated using an electrospinning system (SS-X3, Ucalery, China). Hexafluoroisopropanol (HFIP) and UP water (85:15, v/v) were used as the solvent system. NPs were dispersed in the solvent via 30-min ultrasonication, while PLGA/gelatin/dECM blends were homogenized by magnetic stirring for 4 h. The two solutions were thoroughly mixed to prepare spinning inks with a total solid content of 20% w/v. The mass ratios of components were as follows: NFs (PLGA:gelatin = 1:1), dECM NFs (PLGA:gelatin:dECM = 3:3:2), MPN@NFs (PLGA:gelatin:NPs = 19:19:2), and MPN@dECM NFs (PLGA:gelatin:dECM:NPs = 7:7:5:1). Electrospinning was performed at ±8 kV using a 20G blunt needle and a high-speed rotary collector (2800 rpm, 10 cm in diameter) positioned 15 cm from the needle tip. A syringe pump delivered a fixed volume of 1.5 mL per batch at a controlled flow rate to maintain uniform membrane thickness and ensure stable jet formation without dripping. The resulting fiber membranes were dried in an ambient-temperature oven for 3 days then stored dry and protected from light at −20 °C.

*Characterization of MPN@dECM NFs*: The microstructure and physicochemical properties of electrospun fibers were systematically characterized. For internal structural analysis, a small quantity of fibers collected on carbon-coated copper grids during electrospinning was examined by TEM (Talos F200X), and Mg distribution (Mg-Kα signal) was mapped via EDS in HAADF-STEM mode. SEM imaging was performed on platinum-sputtered fiber membranes using GeminiSEM 500 (Zeiss) operated at 10 kV. Fiber orientation was quantified from SEM images using the OrientationJ plugin (gradient method: finite difference) in ImageJ (v1.54f, NIH, USA), while fiber diameter distributions were determined by randomly measuring 50 fibers per image, with PDI calculated as (*σ*/*μ*)^2^ (*σ*: standard deviation; *μ*: mean diameter). Porosity was assessed through grayscale thresholding (OTSU algorithm) of SEM images in ImageJ. Hydrophilicity was evaluated via water contact angle measurements using JC2000D3 (Powereach, China) with sessile drop method, where images were captured 2 s after droplet contact. Degradation profiles were monitored by incubating square fiber membranes (≈ 1 cm × 1 cm) in 2 mL pH 6.0 saline at 37 °C, with daily saline replacement, and residual areas were normalized to initial values. For the Mg^2+^ release kinetics study, MPN-containing fiber membranes (50 mg) were placed in pH-adjusted phosphate-buffered saline (PBS, pH 6.0/7.4, 10 mL) and incubated at 37 °C with shaking at 40 rpm. Supernatant was collected every 24 h, centrifuged (16,000 × g, 4 °C, 10 min), diluted 10-fold with dilute HCl, and analyzed for Mg^2+^ quantification via Agilent 5800 ICP-OES. For antioxidant activity, 7 mm-diameter fiber discs were immersed in 2 mL of 0.1 mmol/L DPPH· ethanol solution, sealed to prevent evaporation. Absorbance at 517 nm was measured hourly for the first 8 h, followed by additional measurements at 16 h and 24 h using SpectraMax Plus 384. A standard curve generated from DPPH· concentration gradients was used to calculate scavenging rates, with baseline scavenging defined as 0% at 0 h.

*Cell culture*: HT-22 cells were purchased from Wuhan Servicebio Technology Co., Ltd. BV-2 cells were purchased from Wuhan Pricella Biotechnology Co., Ltd. (China). Both cells were maintained in Dulbecco’s Modified Eagle Medium (DMEM, #11995, Gibco) supplemented with 10% fetal bovine serum (FBS, Gibco) and 1% penicillin/streptomycin (P/S, Gibco) under a humidified atmosphere of 5% CO_2_ at 37 °C in an incubator (CLM-170B-8-NF, Esco, Singapore). Cells were subcultured daily at a 1:3–4 split ratio using 0.25% trypsin–EDTA (Gibco) and passaged 3–5 times prior to experimental use.

*Preparation of conditioned media and scaffold leachates*: Conditioned media were prepared by supplementing complete DMEM with either 4 mmol/L L-glutamate (#G107510, Aladdin) (for excitotoxicity modeling) or 1 μg/mL LPS (#L2880, Sigma-Aldrich) (for inflammatory modeling). Scaffold leachates were generated following ISO 10993-12:2021 guidelines. Briefly, ultraviolet (UV)-sterilized fiber membranes (0.5 h per side) were immersed in pre-warmed conditioned or complete medium at a surface area-to-medium ratio of 6 cm^2^/mL and incubated at 37 °C for 24 h. The leachates were then filtered through 0.22 μm polyethersulfone syringe filters (#SLGP033N, Millipore, USA) to remove particulate residues.

*In vitro biocompatibility assay*: HT-22 cells were seeded in 96-well plates at 2000 cells/well and cultured for 24 h. The medium was then replaced with fresh complete medium (control group) or leachate (scaffold groups), with subsequent medium/leachate renewal every 24 h. Cell viability was assessed at 1, 3, and 5 days post-treatment using a CCK-8 assay (#abs50003, Absin, China). Briefly, 110 μL of CCK-8 reagent (1:10 diluted in DMEM) was added to each well, followed by 2 h incubation under standard culture conditions (37 °C, 5% CO_2_). Absorbance at 450 nm was measured using SpectraMax Plus 384 microplate reader.

*Cytoskeletal staining*: The UV-sterilized fiber membranes were immersed in complete medium and incubated in a CO₂ incubator at 37 °C for 1 h. Following the removal of the medium, HT-22 cells were seeded onto the membranes at a density of 5000 cells per cm^2^ and cultured for 12 h. The membranes were then washed twice with PBS, followed by fixation with 4% PFA and permeabilization with 0.3% Triton X-100. Staining was performed by incubating the samples with a working solution containing 100 nM Phalloidin-FITC (#BL1188A, Biosharp, China) and 1 μg/mL Hoechst 33342 (#AG51012, AG Accurate, China) at room temperature for 30 min. After three washes with PBS, fluorescence imaging was carried out using Olympus APX100.

*In vitro excitotoxicity inhibition assay*: The neuroprotective effects of fibrous scaffolds against glutamate-induced excitotoxicity were assessed using HT-22 cells. Cells were seeded at densities of 5000 cells/well in 96-well plates, 25 000 cells/well in 24-well plates, or 25 000 cells/dish on glass-bottom confocal dishes (15 mm diameter). After 24 h of culture, the medium was replaced with the following treatments: control group (complete medium), L-glutamate group (L-glutamate-conditioned medium), or scaffold groups (leachates prepared in L-glutamate-conditioned medium). For 96-well plates, cell viability and cytotoxicity were evaluated at 24 h post-treatment using CCK-8 assay and LDH release assay, respectively. In 24-well plates, cells were stained at 6 h post-treatment with Calcein-AM/PI (live/dead assay), DCFH-DA (ROS detection), and JC-1 (mitochondrial membrane potential). Additional Calcein-AM/PI staining was performed at 24 h. For confocal dishes, intracellular Ca^2+^ flux was analyzed at 6 h post-treatment using Fluo-4 AM staining.

LDH assay was performed using a commercial kit (#G1610, Servicebio). For extracellular LDH release, culture supernatants (80 μL) were collected after centrifugation (1000 × g, 5 min). For total intracellular LDH content, cells were lysed with 120 μL lysis buffer (37 °C, 45 min), and lysate supernatants (80 μL) were collected after centrifugation (1000 × g, 5 min). Absorbance at 490 nm was measured using SpectraMax Plus 384 after incubating samples with LDH working reagent (1:1 v/v) at 37 °C for 30 min under light-protected conditions.

For all fluorescence staining assays, cells were rinsed twice with pre-warmed DMEM (37 °C), incubated with specific working solutions in a humidified 5% CO_2_ atmosphere at 37 °C under light-protected conditions, and washed three times with 37 °C PBS before immediate fluorescence imaging using Olympus APX100. Quantitative analysis of fluorescence intensity was performed based on grayscale values using ImageJ software. Live/dead cell staining was performed with a Calcein-AM/PI kit (#G1707, Servicebio, China), incubating cells in DMEM-based working solution for 15 min. Intracellular ROS levels were detected using a solution containing 10 μmol/L DCFH-DA (#D6883, Sigma-Aldrich) and 1 μg/mL Hoechst 33342 for 30 min. Mitochondrial membrane potential was analyzed via a JC-1 assay (#G1515, Servicebio), with working solution (1:1 in complete medium) supplemented with 0.05% Pluronic F-127 (#P2443, Sigma-Aldrich) and 1 μg/mL Hoechst 33342 during a 30-min incubation. Intracellular Ca^2+^ flux was monitored using 1 μmol/L Fluo-4 AM (#HY-101896, MedChemExpress) and 1 μg/mL Hoechst 33342 in DMEM for 1 h. All dye concentrations and incubation times were referred to the instructions.

*In vitro immunomodulation assay*: BV-2 cells were seeded in 6-well plates at 200 000 cells/well and cultured for 24 h. The medium was replaced with fresh complete medium (control group), LPS-conditioned medium, or scaffold leachates prepared in LPS-conditioned medium. Cell culture supernatants were collected at 6 h and 24 h for cytokine quantification via ELISA. At 24 h post-treatment, cells were harvested for parallel analyses: (1) RNA extraction and RT–qPCR to assess inflammatory gene expression, and (2) flow cytometry to evaluate surface marker profiles.

Cytokine levels (TNF-α, IL-6, IL-12p70, TGF-β1) in culture supernatants were quantified using commercial ELISA kits (NeoBioscience, China: TNF-α #EMC102a, IL-6 #EMC004, IL-12p70 #EMC006, TGF-β1 #EMC107b). Supernatants were centrifuged (1000 × g, 5 min), diluted as specified (TGF-β1 required acid activation followed by neutralization), and analyzed according to the manufacturer’s protocol. Briefly, pre-coated plates were incubated sequentially with samples/standards, biotinylated detection antibodies, horseradish peroxidase (HRP) conjugates, and 3,3',5,5'-tetramethylbenzidine (TMB) substrate under light-protected conditions (37 °C, incubation times per kit instructions). Absorbance at 450 nm was measured immediately using SpectraMax Plus 384 microplate reader.

Total RNA was isolated from treated cells using RNAiso Plus reagent (#9109, Takara, Japan). Briefly, cells were centrifuged (1000 × g, 5 min), washed with PBS, and lysed with RNAiso Plus. Chloroform was added to separate phases, and the aqueous layer was collected for RNA precipitation with isopropanol. RNA pellets were washed with 75% ethanol, air-dried, and dissolved in DEPC-treated water (#B501005, Sangon, China). RNA concentration and purity were verified using NanoDrop OneC spectrophotometer. Reverse transcription was performed with 1 μg RNA using the reverse transcription Kit (#AG11728, AG Accurate) on Biometra TAdvanced thermal cycler (Analytik Jena, Germany) under the following conditions: 37 °C for 15 min, 85 °C for 5 s, and 4 °C hold. Quantitative PCR (qPCR) was conducted using SYBR Green Master Mix (#AG11701, AG Accurate, China) on CFX96 Real-Time System (Bio-Rad, USA) with 40 amplification cycles. Primer pairs (sequences listed in Table S1) were synthesized by Sangon Biotech (Shanghai) Co., Ltd. GAPDH was selected as the internal reference.

Surface marker expression was analyzed to evaluate cellular inflammatory responses. Briefly, adherent cells were detached using trypsin–EDTA, filtered through a 40 μm cell strainer to remove aggregates, and centrifuged (3000 rpm, 5 min). Cell pellets were resuspended in PBS containing 3% FBS for counting and aliquoted into tubes at 4 × 10^5^ cells/tube. After centrifugation, cells were stained with 100 μL fluorochrome-conjugated antibody cocktails (details in Table S2) or matched isotype controls (IgG2a) under light-protected conditions for 30 min, with periodic gentle mixing. Following two PBS washes, cells were resuspended in 400 μL PBS and analyzed on CytoFLEX LX flow cytometer (Beckman Coulter, USA). Voltage settings were calibrated using isotype controls to position all fluorescence signals below the negative threshold (10^3^). A minimum of 10 000 single-cell events were recorded per sample.

*Laboratory animal sources and ethics*: 7-week-old male C57BL/6 mice used for in vivo experiments were purchased from Shanghai Sinogene Biotechnology Co., Ltd. (China) and housed under specific pathogen-free (SPF) conditions. All animal experimental protocols were approved by the Bioethics Committee of East China University of Science and Technology (Approval No. ECUST-2025-085), in compliance with the National Standard of China (GB/T 35892-2018: Guidelines for Ethical Review of Animal Welfare) and the NIH Guide for the Care and Use of Laboratory Animals (8th Edition, 2011).

*SCI modeling, scaffold implantation and post-surgical management*: All surgical procedures were performed under aseptic conditions with mice anesthetized via intraperitoneal injection of sodium pentobarbital (80 mg/kg). Following dorsal fur shaving and disinfection with 75% ethanol, a 1.5 cm midline incision was made over the T9–T10 vertebrae, and paravertebral muscles were dissected to expose the spinal column. A laminectomy at T9–T10 exposed the spinal cord, after which a right lateral hemisection (≈ 1.5 mm length) was created using a microsurgical blade. UV-sterilized fiber scaffolds were hydrated in sterile saline, manually rolled perpendicular to fiber alignment, trimmed to match the lesion length, and implanted into the spinal cord defect cavity as cylindrical constructs. Sham-operated mice underwent laminectomy without spinal injury, while SCI controls received hemisection without scaffold implantation. Incisions were closed with 4-0 nylon sutures and disinfected with iodophor. Postoperatively, animals were maintained on a 37 °C heating pad until ambulatory, housed in sterile cages with ad libitum access to food and water, and received twice-daily manual bladder expression until spontaneous urination resumed (typically 7–14 days) or endpoint. For mice requiring EdU staining, EdU (#G5059, Servicebio, 1 mg/mL in saline) was administered intraperitoneally at a daily dose of 5 mg/kg from the day of surgery until sacrifice on day 10.

*Motor function evaluation*: Motor functional recovery was longitudinally assessed using the Basso Mouse Scale (BMS) and Inclined Plane Test (IPT) at preoperative day 1, postoperative days (POD) 0 (after full recovery from anesthesia), 1, 3, 5, 7, and weekly thereafter until week 8. For BMS scoring, mice were individually placed in an open-field arena, and their behavior were video-recorded over 4 min. Three trained blinded observers independently scored locomotion of the ipsilateral (right) hindlimb and trunk according to the standardized BMS 9-point scale, with the median score used for analysis. IPT performance was evaluated by determining the maximum incline angle (integer degrees) at which mice maintained upward progression for 5 s on a rubber surface. Starting angles began at 10° on POD 0, then dynamically adjusted: increased by 1° after successful trials or decreased by 1° after failures until the critical angle was identified. Mice rested ≥ 5 min between attempts to prevent fatigue. At week 8, spatiotemporal gait parameters were quantified using the VisuGait Small Animal Gait Analysis System (XR-FP101, XinRuan, China). Mice freely traversed a transparent walkway, and footprints were captured via a high-speed camera integrated with footprint light refraction technology. Automated software generated qualitative and quantitative gait profiles based on paw strike patterns. All behavioral tests were conducted between 9:00–15:00 after 30 min environmental acclimation.

*Tissue Collection and Sample Preparation*: For histological evaluation, mice were euthanized at 1 day, 3 days, 10 days or 8 weeks post-surgery via cervical dislocation under deep isoflurane anesthesia (#R510-22-10, RWD, China), and spinal cords were dissected to retain 3 mm of tissue rostral and caudal to the lesion epicenter. Freshly isolated tissues were rinsed in PBS for 10 min to remove residual blood. Tissues collected on day 1 were weighed and homogenized on ice in PBS at a weight-to-volume ratio of 1:9, followed by centrifugation at 12 000 × g to collect the supernatant for ROS measurement. Tissues harvested on day 3 were gently blotted to remove residual PBS on the surface, immediately embedded in OCT compound, and frozen for cryosectioning into 12 μm-thick slices, which were used for ROS staining. For tissues obtained on day 10, fixation was carried out in 4% PFA at 4 °C overnight, followed by sucrose dehydration, OCT embedding, and sectioning at 10 μm thickness for EdU-immunofluorescence staining. Tissues collected at 8 weeks were fixed in 4% PFA at 4 °C for 48 h, then paraffin-embedded, sectioned into 5 μm slices, baked sequentially at 50 °C for 30 min and 65 °C for 10 min, and finally used for H&E, LFB, Masson’s trichrome, and immunofluorescence staining.

*Measurement of ROS in tissue homogenates*: Fresh tissue homogenate (10 μL) was added to each well of a black 96-well plate with a transparent bottom. Background control wells received 10 μL of PBS instead. Then, 100 μL of 10 μmol/L DCFH-DA was added to each well, followed by incubation at 37 °C for 20 min. Fluorescence was measured using a SpectraMax Plus 384 microplate reader with excitation at 488 nm and emission at 525 nm. The relative fluorescence units (RFU) were quantified after background subtraction.

*ROS Staining*: Tissue sections collected on day 3 were warmed to room temperature, followed by washing in PBS to remove OCT. Then, 10 μmol/L DCFH-DA was applied to the sections and incubated at 37 °C for 30 min. After incubation, the samples were washed three times with PBS. Finally, the sections were mounted with the anti-fade mounting medium with DAPI.

*LFB staining*: LFB staining was performed using a commercial kit (#G1030, Servicebio). Briefly, slides were dewaxed in xylene (2 × 20 min), rehydrated through a graded ethanol series (100% to 75%), and stained with pre-warmed LFB solution (60 °C, 30 min activation) at 60 °C for 3–4 h. After cooling and rinsing, differentiated in 70% ethanol and lithium carbonate solution alternately until optimal myelin contrast was achieved under microscopic monitoring. Slides were dehydrated through absolute ethanol (3 × 5 min), cleared in xylene (2 × 5 min), and mounted with neutral resin for myelin visualization.

*Masson’s trichrome staining*: Masson’s trichrome staining was performed using a commercial kit (#G1340, Solarbio). Briefly, slides were dewaxed in xylene (2 × 20 min) and rehydrated through a graded ethanol series (100% to 75%). After overnight immersion in potassium dichromate solution and brief rinsing, slides were stained with iron hematoxylin (3 min), differentiated in acid alcohol, and counterstained with ponceau-acid fuchsin (5–10 min). Tissues were treated with phosphomolybdic acid (1–3 min), directly stained with aniline blue (3–6 min) without washing, and differentiated in 1% acetic acid. Following dehydration in ethanol (2 × 5 min) and clearing in xylene (5 min), slides were mounted with neutral resin for microscopic analysis.

*Immunofluorescence staining*: Immunofluorescence staining was performed using antibodies listed in Table S2. Slides were dewaxed in xylene (3 × 5–10 min), rehydrated through graded ethanol (100% to 70%), and subjected to antigen retrieval in citrate buffer (pH 6.0) via microwave irradiation (high power 3 min, medium power 3 min, low power 1 min; 5 min intervals). Then the slides were blocked with 5% serum (matched to secondary antibody host species) at 37 °C for 30 min, incubated with primary antibody cocktails (diluted in PBST) at 4 °C overnight, rewarmed for 15 min, and washed (4 × 5 min PBST). Fluorescent secondary antibody cocktails were applied (1 h, 37 °C, light-protected), then the slides were washed (4 × 5 min PBST) and mounted with the anti-fading mounting medium with DAPI.

*EdU staining*: After thawing and removing OCT from the frozen sections, the samples were blocked with 5% serum at room temperature for 30 min. Subsequently, immunofluorescence staining was performed by incubating with primary and secondary antibodies, followed by washing with PBST. The sections were then incubated with Click-iT EdU-555 (#G1602, Servicebio) reaction cocktail at room temperature for 30 min, washed again with PBST, and finally mounted using the anti-fading mounting medium with DAPI.

All stained slides were imaged using Olympus APX100 slide scanner, digitally processed with OlyVIA software, and subsequently analyzed in ImageJ for quantitative analysis. For immunofluorescence images of the tissue sections, a consistent positivity threshold was applied uniformly across all images for each specific marker to quantify the positive area within defined regions of interest.

*Statistical analysis and data visualization*: Data were analyzed using Origin (v10.1.5.132) or GraphPad Prism (v9.5.1, GraphPad Software, USA) and expressed as mean ± standard deviation (SD) from at least three parallel samples (*n* ≥ 3). Statistical significance was determined by Student’s t-test (two-group comparisons), one-way ANOVA (three or more groups), or two-way ANOVA (multiple variables), followed by Tukey’s post hoc test as specified in figure captions. A threshold of *p* < 0.05 was considered statistically significant. All reported trends were validated through three independent experimental replicates. Schematic diagrams and figures were assembled and annotated in Adobe Illustrator (v25.0, Adobe Systems, USA).

4. Supplemental Tables

**Table S1.** Primer sequences for RT-qPCR.

| Primer | Forward (5' to 3') | Reverse (5' to 3') |
| --- | --- | --- |
| GAPDH | TGAACGGGAAGCTCACTGG | GCTTCACCACCTTCTTGATGTC |
| IL6 | CAACGATGATGCACTTGCAGA | TGTGACTCCAGCTTATCTCTTGG |
| IL1β | TGCCACCTTTTGACAGTGATG | AAGGTCCACGGGAAAGACAC |
| TGFβ1 | ACTGGAGTTGTACGGCAGTG | GGGGCTGATCCCGTTGATT |
| iNOS | TCTAGTGAAGCAAAGCCCAACA | TGATGGACCCCAAGCAAGAC |
| Arg1 | ATCGGAGCGCCTTTCTCAAA | CTTCCAACTGCCAGACTGTG |
| Ly6C | TGCCTGCAACCTTGTCTGAG | TGCAGTCCCTGAGCTCTTTC |

**Table S2.** Antibodies.

| Name | Cat. No. | Brand | Application | Dilution |
| --- | --- | --- | --- | --- |
| FITC-conjugated Anti-CD86 Rat Mab | 105005 | Biolegend | Flow-cyto | 1:100 |
| APC-conjugated Anti-CD206 Rat Mab | 141707 | Biolegend | Flow-cyto | 1:100 |
| FITC-conjugated Rat IgG2a | 400505 | Biolegend | Flow-cyto | 1:100 |
| APC-conjugated Rat IgG2a | 400511 | Biolegend | Flow-cyto | 1:100 |
| Anti-Iba1 Goat Rec Mab | ab289874 | Abcam | Immunofluorescence | 1:1000 |
| Anti-TUBB3 Rabbit Rec Mab | 80713-1-RR | Proteintech | Immunofluorescence | 1:500 |
| Anti-NeuN Rat Rec Mab | ab279297 | Abcam | Immunofluorescence | 1:1000 |
| Anti-GAP43 Rabbit Rec Mab | A19055 | ABclonal (China) | Immunofluorescence | 1:1000 |
| Anti-MBP Rat Rec Mab | ab7349 | Abcam | Immunofluorescence | 1:1000 |
| Anti-GFAP Rabbit Rec Mab | A19058 | ABclonal (China) | Immunofluorescence | 1:1000 |
| Alexa Fluor 488-conjugated Donkey Anti-Goat IgG (H+L) | ab150129 | Abcam | Immunofluorescence | 1:500 |
| Alexa Fluor 568-conjugated Donkey Anti-Rat IgG (H+L) | ab175475 | Abcam | Immunofluorescence | 1:500 |
| Alexa Fluor 647-conjugated Donkey Anti-Rabbit IgG (H+L) | ab150075 | Abcam | Immunofluorescence | 1:500 |
